# Combined metformin and simvastatin therapy inhibits SREBP2 maturation and alters energy metabolism in glioma

**DOI:** 10.1038/s41419-024-07169-5

**Published:** 2024-11-09

**Authors:** Xiaolong Qiao, Zixuan Wang, Yinan Chen, Nan Peng, Hongwei Zhang, Chaoshi Niu, Chuandong Cheng

**Affiliations:** 1 Department of Neurosurgery, Centre for Leading Medicine and Advanced Technologies of IHM, The First Affiliated Hospital of USTC, Division of Life Sciences and Medicine, University of Science and Technology of China, Hefei, Anhui 230001 China; 2 Anhui Universitie of Science and Technology, Huainan, Anhui 232001 China

**Keywords:** CNS cancer, Target validation

## Abstract

This study aims to explore the inhibitory effects of combined metformin and simvastatin therapy on the malignant progression of glioma. The research specifically examines how the maturation of SREBP2 as a transcription factor affects the expression of GLUT1 and GLUT6 in glioma cells. Additionally, it investigates the impact of this combination therapy on the biological functions and energy metabolism of glioma cells. To assess the functions of GLUT1/6, sh-GLUT1/6 plasmids were employed. The study determined the half-maximal inhibitory concentrations (IC50) of metformin and simvastatin using the CCK-8 assay. Subsequently, the effects of these drugs on glioma metabolism, proliferation, and apoptosis were explored in vitro and in vivo, using drug concentrations significantly lower than their respective IC50 values. The impact of drug treatment on GLUT1/6 and SREBP2 expression levels was also evaluated. The study elucidated the significant impact of GLUT1/6 on glioma cell functions, resulting in decreased glucose uptake. Moreover, it unveiled the regulatory role of SREBP2 in GLUT1 and GLUT6 transcription, alongside revealing differential expression of SREBP2 precursor and mature forms within gliomas. Following combined drug therapy, GLUT1/6 expression decreased, while the precursor form of SREBP2 increased, and mature SREBP2 reduced. This dual-drug treatment effectively modulated glioma cell energy metabolism. Subsequent in vivo experiments affirmed the augmented anti-tumor efficacy of combined drug therapy. Specifically, the synergistic action of metformin and simvastatin reshaped glioma metabolism, curbed malignant proliferation, promoted apoptosis, and demonstrated superior anti-tumor effects both in vitro and in vivo compared to individual administration of metformin or simvastatin. Importantly, the combination therapy achieved these effects at lower doses, rendering it a safer treatment option.

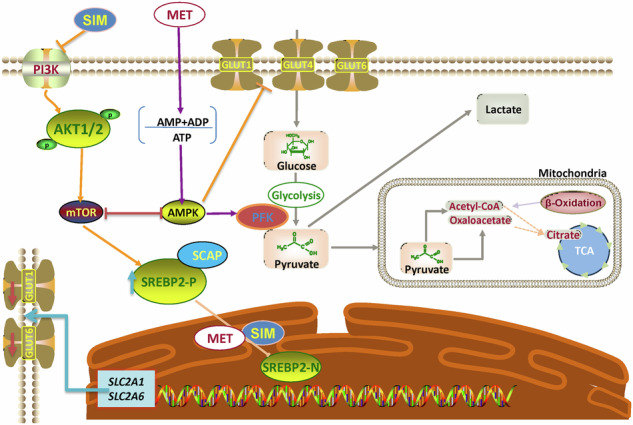

## Introduction

Glioma stands as the most prevalent malignant primary brain tumor among adults. Glioblastoma (GBM), classified as a WHO IV glioma, is marked by its dismal prognosis, extensive tumor heterogeneity, and the absence of effective treatments, boasting a mere 5-year survival rate of 7.2% [1, 2]. Despite two decades of intensive research, all clinical trials during this period have failed to enhance outcomes for GBM patients. Surgical resection remains challenging due to the aggressive nature of GBM and the difficulty in defining their margins [3]. Sterol Regulatory Element-Binding Protein 2(SREBP2), initially an Endoplasmic Reticulum (ER) membrane protein bound to SREBP cleavage-activating protein (SCAP), moves to the Golgi apparatus when cholesterol levels drop. There, proteases Site-1 protease (S1P) and Site-1 protease (S2P) cleave SREBP2 [4], releasing its N-terminal portion—a mature transcription factor. This mature form relocates to the nucleus to regulate genes related to cholesterol and lipid synthesis [5]. Our previous work has demonstrated that SREBP2 regulates GLUT1 and GLUT6 at the transcriptional level, while the expression of other GLUT family members in the brain of glioma patients is not specific [6]. The relationship between Glucose Transporters (GLUTs) and cancer has attracted widespread attention in research [7]. GLUTs are a class of membrane proteins responsible for transporting glucose from the extracellular environment into the cells, providing energy and supporting growth and development [8]. In cancer cells, the expression levels of GLUTs are often significantly elevated; enabling cancer cells to more effectively acquire glucose and meet their energy demands for rapid growth [9].

In glioma and other tumors, the Warburg effect plays a crucial role in supporting rapid cell proliferation and tumor growth [10, 11]. The phenomenon has become a significant area of research in cancer metabolism and represents a potential target for anti-cancer therapies. Fuentes-Fayos et al. explored the significance of the combined effects of metformin and simvastatin in glioblastoma, demonstrating that these drugs exert additive antitumor effects through mechanisms related to senescence [12]. Metformin is a commonly used oral hypoglycemic medication primarily prescribed for treating type 2diabetes. Recent research indicates that Metformin can penetrate the blood-brain barrier (BBB) and enter brain tissues [13]. In GBM studies, Metformin is believed to potentially exert its anticancer effects by regulating cellular metabolism, influencing tumor cell proliferation, apoptosis, and other pathways [14, 15]. Experimental evidence from in vitro and in vivo studies has demonstrated its inhibitory effects on the malignant progression of gliomas [16, 17]. Metformin can activate the AMPK pathway, influencing the downstream AKT pathway to regulate subsequent molecules [18]. Recent studies have shown that simvastatin has a promising anti-tumor effect in gliomas [19, 20]. Simvastatin is a beta-hydroxy-beta-methylglutaryl-coenzyme A (HMG-CoA) reductase inhibitor primarily used to lower cholesterol levels. Research has shown that simvastatin can indirectly inhibit the mTOR pathway [21], activate the AMPK pathway [22], and modulate the SREBPs pathway [23].

Our study investigates how the combined use of metformin and simvastatin affects glioma progression by modulating glucose metabolism and SREBP2 signaling. We aimed to determine whether these drugs could work together to inhibit glioma growth through the regulation of key glucose transporters (GLUT1 and GLUT6) and their associated regulatory pathways. Our findings demonstrate that the combination therapy significantly reduces glioma cell proliferation and alters metabolic pathways, providing new insights into potential therapeutic strategies for glioma treatment.

## Materials and methods

### Patients and tissue samples

In this study, a total of 102 patients diagnosed with gliomas (*n* = 102) who underwent initial surgical resection at The First Affiliated Hospital of the University of Science and Technology of China between 2020 and 2022 were included. Among these patients, 21 had low-grade gliomas (LGG) and 81 had high-grade gliomas (HGG). Additionally, 20 normal tissue samples were collected from patients undergoing temporal lobectomy due to temporal lobe epilepsy.

Prior to the surgical procedure, informed consent was obtained from each patient, and the use of samples for this research was approved by The Medical Ethics Committee of The First Affiliated Hospital of the University of Science and Technology of China. It is worth noting that all patients included in the study were treatment-naive before the surgical resection. Further detailed information about the patients and samples can be found in Table S1.

### Cell lines and cell culture

In this study, various cell lines were utilized. U87, U251, LN229, T98G, and TJ905, all representing glioblastoma (GBM), were procured from the American Type Culture Collection (ATCC, Manassas, VA, USA). Additionally, HEB, derived from normal human astrocytes, was also obtained from ATCC. These cell lines were cultured in Dulbecco’s Modified Eagle Medium (DMEM; Gibco, Carlsbad, CA, USA) and maintained at a temperature of 37 °C in a humidified atmosphere with 5% CO_2_. The culture medium was supplemented with 10% fetal bovine serum (Gibco, NY, USA) and 5% penicillin-streptomycin-gentamicin antibiotics (Solarbio, Beijing, China). This standardized culture condition ensured the appropriate growth and maintenance of the cell lines throughout the experimental procedures.

### Immunohistochemistry

Protein expression was performed by IHC. The primary antibodies used in this study and their respective dilutions were as follows: GLUT1 (1:200), GLUT6 (1:200), SREBP2 (1:200), and Ki-67 (1:500). The sections were then counterstained with hematoxylin. Specific procedures are described in the supplementary material.

### IC50 Determination

Metformin was dissolved in PBS to a 20 mM concentration and filtered, while Simvastatin was dissolved in DMSO as a 1 mM working solution. Cells (2000 cells/well) were seeded in a 96-well plate and treated with varying Metformin concentrations (0–40 mM) and specific Simvastatin concentrations (0-50 μmol) for 48 h. Following treatment, CCK-8 solution was added, and cells were incubated at 37 °C for 1 h. IC50 curves were generated using GraphPad Prism 9 software.

### Mitochondrial membrane potential assessment

Mitochondrial membrane potential was determined using Tetrechloro-tetraethylbenzimidazol carbocyanine iodide (JC-1), following the protocols provided by the respective manufacturers (Beyotime, China). Pretreated cells were washed with PBS, followed by incubation with 1 ml of JC-1 working solution per sample at 37 °C for 20 minutes. After incubation, cells were washed, cover-slipped with neutral balsam, and illuminated with 488 nm excitation. Both green and red fluorescence signals were recorded, and image analysis was performed using NIS-Elements software (Nikon CEE GmbH, C.R.) for evaluation.

### Alkaline comet assay (Slide Method)

Cell damage was evaluated using the Comet electrophoresis assay kit, following protocols from the respective manufacturers (Jiangsu KeyGEN BioTECH Corp, China). Microscope slides were prepared by dipping in 0.9% agarose, air-drying, and covering with a cell/agarose suspension. After lysis, slides underwent electrophoresis, neutralization, and propidium iodide staining. The slides were then stored in a humidified container and analyzed within 24 h. This streamlined procedure enables efficient assessment of DNA damage.

### Determining ATP content, lactic acid content, and pyruvate content

ATP Content, Lactic Acid Content, and Pyruvate Content were measured using specific assay kits. The ATP level was determined using the ATP Assay Kit from Beyotime, China, following the manufacturer’s protocol. Lactic Acid Content was evaluated using the Lactic Acid (LA) HPLC Assay Kit from Solaibao, China, according to the provided instructions. Pyruvate Content was assessed using the Pyruvate (PA) Content Assay Kit from Solaibao, China, following the manufacturer’s guidelines. These assays are essential for studying cellular energy metabolism and various metabolic processes.

### Drugs

Metformin Hydrochloride obtained from Solarbio (Beijing, China) was dissolved in phosphate-buffered saline (PBS) to create a stock solution of 200 mM. Simvastatin purchased from MedChemExpress (NJ, USA) was dissolved in Dimethyl Sulfoxide (DMSO) to create a stock solution of 1 mM. Betulin obtained from MedChemExpress (NJ, USA) was dissolved in DMSO to create a stock solution of 1 mM. In the cell experiments, metformin was used at a concentration of 1 mM, simvastatin was used at 2 µM, and when combined, the concentrations were 0.5 mM for metformin and 1 µM for simvastatin. Additionally, Betulin was used at a concentration of 6 µM.

### Seahorse analysis

The mitochondrial bioenergetics of HEB, U87, and T98G cells, in response to metformin and simvastatin, were assessed using a Seahorse XFe96 Analyzer (Agilent Technologies, USA). Cells were seeded at a density of 2 × 10^4^ per well in 96-well transparent plates 24 h before treatment. After a 48-hour incubation with the drugs, the medium was replaced with Seahorse XF base medium (Agilent Technologies, USA) and incubated for 1 h at 37 °C in a CO_2_-free environment.

Subsequently, the following chemicals were injected into the medium to measure oxygen consumption rate (OCR): (a) oligomycin (OM; 10 mM), an ATP synthase inhibitor; (b) p-trifluoromethoxy carbonyl cyanide phenylhydrazone (FCCP; 10 mM), an uncoupler that maximizes respiratory rate; (c) a mixture of rotenone (ROT) and antimycin A (AA) (50 mM), which inhibit mitochondrial complexes I and III, respectively. For the extracellular acidification rate (ECAR) assay, the following reagents were sequentially added: (a) glucose (10 mM), which increases glycolysis; (b) oligomycin (OM; 10 mM), an ATP synthase inhibitor; (c) 2-deoxyglucose (2-DG, 50 mM), a competitive inhibitor of hexokinase. Seahorse Wave software was used to analyze the results. ECAR was measured in mpH/min, respectively.

### Animal model

U87 cells (1 × 10^6^) stably expressing MCS-firefly luciferases for bioluminescence imaging were transfected with lentivirus expressing control. These cells were injected into the frontal lobe of nude mice to generate GBM (*n* = 5 mice per group). Prior to injection, the mice were randomly assigned to experimental groups using a computer-generated random number sequence to ensure unbiased allocation. Metformin (200 mg/kg/day) and Simvastatin (200 μg/kg/day) were provided in drinking water.

#### Inclusion and exclusion criteria

Mice were included in the study if they were healthy, of a specific age range (e.g., 6–8 weeks old), and exhibited no signs of pre-existing health conditions. Exclusion criteria included any mice that showed signs of illness or distress prior to treatment, those with body weight below a certain threshold, and any that did not develop tumors by day 7 post-implantation. Tumor volumes were measured using a bioluminescence imaging system on days 7 and 14 after implantation. The integrated flux of photons (photons/s) within each region was determined using the Living Images software package (Caliper Life Sciences). Mice were euthanized when they reached a state of deep coma. Brains were extracted, fixed in 10% formalin, and then embedded in paraffin for IHC or frozen at −80 °C for western blot or qPCR assays. All animal studies were conducted with the approval of the ethics committee of USTC.

#### Blinding Statement

In this study involving animal models, the investigator was not blinded to group allocation during the experiments or when assessing outcomes. While no blinding was implemented, all measures were taken to minimize bias in data collection and analysis.

## Statistical Analysis

All statistical analyses were performed with the SPSS 20.0 statistical software package. Data are expressed as mean ± SD from at least three independent experiments. Differences were evaluated by Student’s t test for two groups, one-way analysis of variance for multiple groups, and parametric generalized linear model with random effects for tumor growth. *P* values < 0.05 were considered statistically significant and all statistical tests asterisks indicate statistical significance.

## Results

### The expression levels of the GLUTs family and the SREBP2 gene and protein in gliomas

**Fig. 1:**
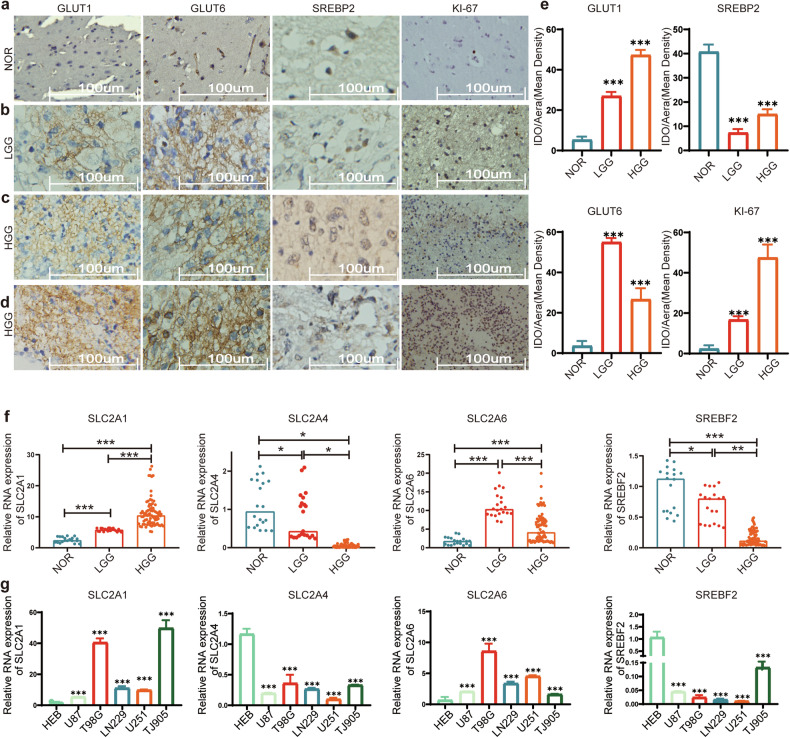
Evaluation of GLUTs and SREBP2 Protein and Nucleic Acid Levels. **a**–**d** Immunohistochemical analysis reveals altered protein expression in normal brain tissues, low-grade gliomas, and high-grade gliomas. It demonstrates elevated levels of GLUT1, GLUT6, and KI-67, alongside reduced expression of SREBP2 in glioma. **e** The immunohistochemical staining intensity was automatically calculated using Image-Pro plus 6.0 to determine the positive rate, and a quantitative map was generated using GraphPad Prism 7. **f** The expression levels of SLC2A1, SLC2A4, SLC2A6, and SREBF2 were analyzed using RT-qPCR in 30 normal tissues, 21 low-grade glioma tissues, and 81 high-grade glioma tissues. **g** RT-qPCR analysis assessed the relative expression levels of SLC2A1, SLC2A4, SLC2A6, and SREBF2 in five glioma cell lines and the HEB normal glial cell line. SLC2A1, SLC2A4, SLC2A6, and SREBF2 encode the proteins GLUT1, GLUT4, GLUT6, and SREBP2, respectively. All experiments were conducted in triplicate, and the data are expressed as the mean ± standard deviation. Statistical significance is denoted by *P < 0.05, **P < 0.01, and ***P < 0.001, as determined by a two-tailed Student’s t-test.

Immunohistochemistry results indicate high expression of GLUT1, GLUT6, and Ki-67 in gliomas, while SREBP2 is expressed at a low level. Among them, GLUT1 increases with tumor grade, showing elevated expression. GLUT6 exhibits the highest expression in low-grade gliomas. SREBP2 expression gradually decreases with tumor progression (Fig. 1a–d).

In this study, 30 samples of normal brain tissues were used as controls. The expression levels of GLUT1, GLUT4, GLUT6, and SREBP2 were validated in 21 cases of low-grade gliomas and 81 cases of high-grade gliomas at the tissue level. It was observed that GLUT1 expression increased with the malignancy of tumors; GLUT4 expression decreased gradually with tumor progression; GLUT6 showed the highest expression in low-grade gliomas, with expression levels in tumor tissues higher than those in normal brain tissues; SREBP2 expression decreased gradually with tumor progression (Fig. 1f). The expression of GLUT1 and GLUT6 increased, while GLUT4 and SREBP2 decreased in glioma cell lines, confirming the gene expression patterns observed in the tumor tissues (Fig. 1g).

### SREBP2 transcriptionally regulates GLUT1 and GLUT6, with differential expression between mature and precursor forms of SREBP2

**Fig. 2:**
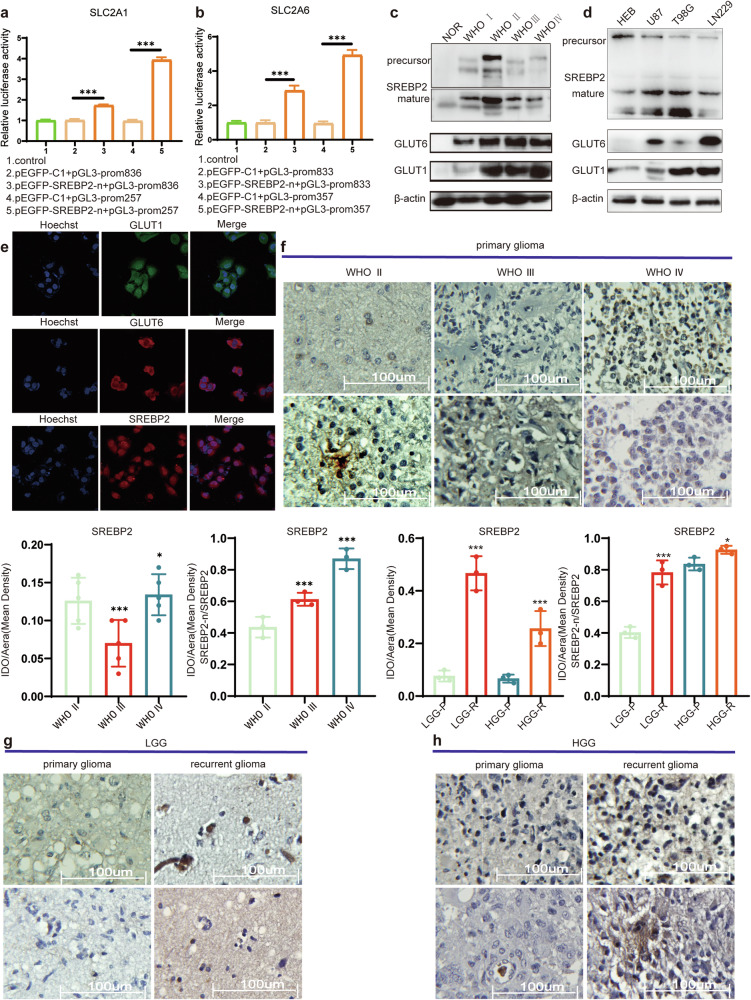
SREBP2: Regulator ofSLC2A1/6 Transcription and Activation Form. **a**, **b** Dual-luciferase reporter gene assay results demonstrate SREBP2 transcriptional regulation of SLC2A1/6. **c**, **d** Expression levels of GLUT1, GLUT6, and SREBP2 precursor and mature forms were assessed through Western blot analysis in both tissues and cell lines. **e** Immunofluorescence staining was performed to assess the intracellular localization of GLUT1, GLUT6, and SREBP2 in U87 cells. **f** IHC detects the expression levels of SREBP2 precursor and mature forms in gliomas of different grades. **g**, **h** IHC detects the expression levels of SREBP2 precursor and mature forms in primary and recurrent gliomas of different grades. SLC2A1 and SLC2A6 encode the proteins GLUT1 and GLUT6, respectively. Statistical significance is denoted by *P < 0.05, and ***P < 0.001, as determined by a two-tailed Student’s t-test.

In our previous study [6], we found that SREBP2 transcriptionally regulates *SLC2A1* and *SLC2A6* (The genes encoding GLUT1 and GLUT6), and the results from the Dual-Luciferase reporter gene assay are consistent with those findings (Fig. 2a, b). More importantly, in this study, we discovered that the changes in the protein levels of GLUT1 and GLUT6 are consistent with the role of mature SREBP2 as a transcription factor (Fig. 2c, d). In glioma tissues, as the malignancy increases, we observed the following: the expression levels of mature SREBP2, GLUT1, and GLUT6 proteins all show an increasing trend. However, the expression level of SREBP2 precursor decreases. Immunofluorescence results demonstrate that GLUT1 and GLUT6 are mainly localized on the cell membrane, confirming the primary function of glucose transporters on the cell membrane. SREBP2 is primarily located in the cytoplasm (Fig. 2e).

To further clarify the expression of SREBP2 precursor and mature forms, we conducted immunohistochemical (IHC) staining analysis on different grades of gliomas. While there were differences in the overall levels of SREBP2 among gliomas of different grades, there was no clear pattern observed (Fig. 2f). However, with the malignancy grade of gliomas increasing, we observed a proportional rise in the mature SREBP2 (SREBP2-n) compared to the total SREBP2 levels (Fig. 2f). Furthermore, we compared the expression of SREBP2 in primary and recurrent gliomas of different grades and found that regardless of the grade, both in high and low-grade gliomas, the overall levels of SREBP2 as well as the proportion of SREBP2-n were increased in recurrent gliomas (Fig. 2g, h).

### The biological functions of GLUT1 and GLUT6 in glioblastoma

**Fig. 3:**
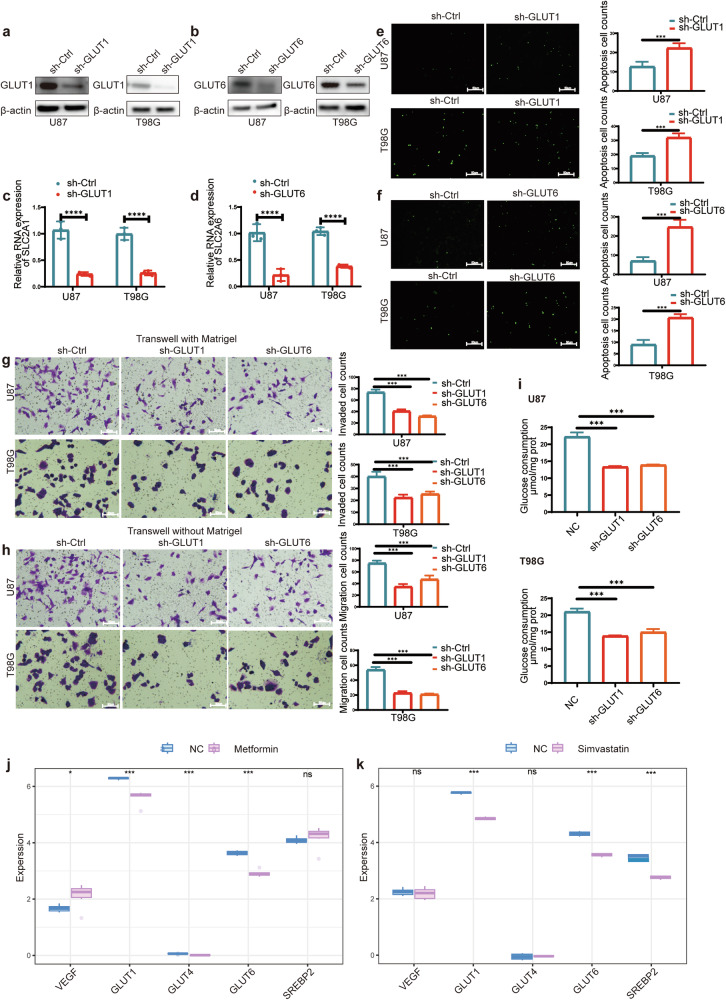
Effects of GLUT1/6 on Biological Functions and Glucose Transport in Glioblastoma Cells. **a**, **b** Western blot analysis determined the relative expression levels of GLUT1/6 following transfection with sh-GLUT1 or sh-GLUT6. **c**, **d** RT-qPCR analysis evaluated the relative expression levels of SLC2A1/6 following transfection with sh-GLUT1 or sh-GLUT6. **e**, **f** The one-step TUNEL assay highlighted an increase in apoptosis within the sh-GLUT1 or sh-GLUT6 group. **g**, **h** The Transwell assay results indicated a significant reduction in the migration and invasion abilities of U87 and T98G cells following transfection with sh-GLUT1 or sh-GLUT6. **i** Quantification of intracellular glucose content in U87 and T98G cells after transfection with sh-GLUT1 or sh-GLUT6. **j**, **k** Analysis of sequencing data after metformin or simvastatin treatment: Validation of the correlation between metformin or simvastatin treatment and the expression of GLUTs and SREBP2. All experiments were conducted in triplicate, and the data are expressed as the mean ± standard deviation. Statistical significance is denoted by ***P < 0.001 and ****P < 0.001, as determined by a two-tailed Student’s t-test.

We designed sh-GLUT1 and sh-GLUT6 plasmids for transfection into U87 and T98G cells. Subsequently, we conducted validation at both the nucleic acid and protein levels, confirming a reduction in GLUT1 and GLUT6 expression (Fig. 3a–d).Transfection of U87 and T98G cells with sh-GLUT1 and sh-GLUT6 plasmids was performed, and one-step TRUNAL experiments revealed that the decrease in GLUT1 or GLUT6 expression led to an increase in apoptosis in glioblastoma cells (Fig. 3e, f). The decreased expression of GLUT1 or GLUT6 resulted in a reduction in the migratory and invasive capabilities of glioblastoma cells (Fig. 3g, h). Most importantly, upon confirming the decreased expression of GLUT1 or GLUT6, we observed a reduction in glucose uptake by the tumor cells (Fig. 3i).

### Bioinformatics analysis reveals the roles of metformin and simvastatin in the energy metabolism of glioblastoma

GBM vary significantly in their genetics and appearance, leading to challenges in developing comprehensive treatments. Recognizing this diversity is vital for creating personalized therapies for glioma patients [24]. In recent years, research targeting the Warburg effect for glioma treatment has been on the rise [25]. Additionally, drugs like metformin and simvastatin, which can penetrate the blood-brain barrier and alter metabolic pathways, have shown great feasibility in the treatment of gliomas. The study utilized sequencing data from previous research on metformin [26] and simvastatin [27]. Analyzing the expression of genes related to tumor energy metabolism, it was found that proteins associated with glucose transport were significantly downregulated after metformin treatment. However, SREBP2, a protein related to cholesterol metabolism, remained unaffected (Fig. 3j). After simvastatin treatment, some Gluts expressions were downregulated and SREBP2 expression was significantly reduced (Fig. 3k). In addition, we analyzed the expression of GLUTs family-related genes, cholesterol metabolism-related genes, as well as VEGF using online databases, and conducted survival prognosis analysis for the patients. GLUT1 and GLUT6 are significantly overexpressed in GBM and associated with poor prognosis, while GLUT4 expression and prognosis show no statistically significant correlation. SREBP2 is downregulated in GBM and is associated with poor prognosis. Additionally, high expression of VEGF in GBM is correlated with poor prognosis (Fig. S1a and S1b).

### Half-maximal inhibitory concentration (IC50) of Metformin and Simvastatin

In our previous study, we found that metformin exhibited inhibitory effects on glioblastoma proliferation both in vitro and in vivo. However, due to experimental constraints, the concentration of metformin used was relatively high, making it challenging to apply in clinical treatments for patients [28]. In this study, we systematically determined the IC50 values of glioblastoma cell lines for metformin and performed fitting calculations (Table S3). We observed that the IC50 values of glioblastoma cell lines were lower than those of HEB **(**Fig. S2a and S2b**)**. In contrast, for simvastatin, the IC50 value in HEB was significantly higher than that in glioblastoma cell lines (Fig. S2c and S2d).

### The Effects of Metformin and Simvastatin on the Proliferation and Apoptosis of glioma Cells

**Fig. 4:**
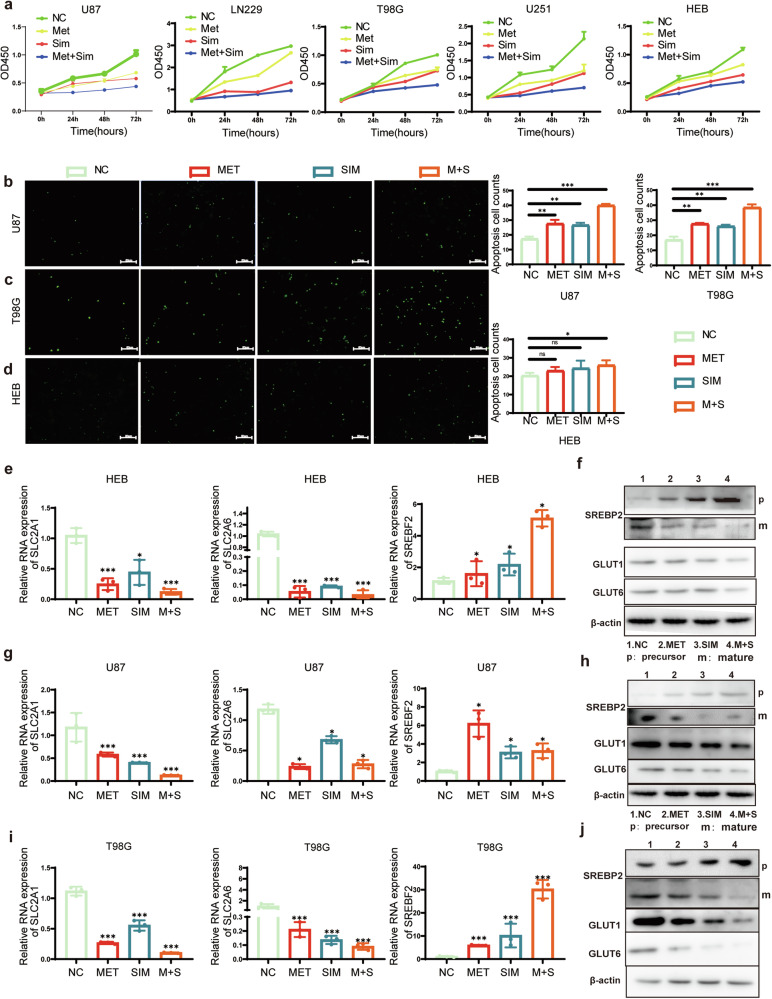
the Effects of Metformin and Simvastatin on Proliferation, Apoptosis, GLUTs, and SREBP2. **a** Cell viability of U87, LN229, T98G, U251, and HEB cells was evaluated using the CCK8 assay with low concentrations of metformin, simvastatin, or their combination treatment (*n* = 3); **b**–**d** The one-step TUNEL assay was utilized to assess apoptosis. **e**, **g**, **i** RT-qPCR assays were employed to assess the mRNA levels of SLC2A1, SLC2A6, and SREBF2 in HEB, U87, and T98G cells treated with different drug concentrations; **f**, **h**, **j** Western blot assays were conducted to examine the protein levels of GLUT1, GLUT6, SREBP2 precursor and SREBP2 mature form in HEB, U87, and T98G cells. For **a**, the drug concentrations are provided in Supplementary Materials Table S3. For **b**–**i**, metformin was used at a concentration of 1 mM, simvastatin at 2 µM, and in combination, the concentrations were 0.5 mM for metformin and 1 µM for simvastatin. All experiments were conducted in triplicate, and the data are expressed as the mean ± standard deviation. Statistical significance is denoted by *P < 0.05, **P < 0.01, and ***P < 0.001, as determined by a two-tailed Student’s t-test.

After 48 h of treatment with low doses of metformin, simvastatin, and their combination, it was observed that the combined treatment had a higher inhibitory effect on the proliferation of U87, LN229, T98G, and U251 glioblastoma cells compared to individual treatments with metformin or simvastatin alone. Additionally, the combined treatment also affected HEB cells, although the tolerance of HEB cells to the drugs was higher compared to glioblastoma cell lines (Fig. S2e). CCK-8 assay was performed to assess the effects of metformin and simvastatin on cell proliferation after 24 h, 48 h, and 72 h of treatment. Results showed that both metformin and simvastatin alone were capable of inhibiting cell proliferation. Moreover, the combined use of these two drugs exhibited the most significant inhibitory effect on glioblastoma cell proliferation (Fig. 4a). Metformin alone promoted apoptosis in U87 and T98G cells, but had no significant effect on HEB cells. Simvastatin alone, as well as in combination with metformin, induced apoptosis in U87, T98G, and HEB cells (Fig. 4b–d).

### The effects of low-dose metformin, simvastatin, and their combination on the expression of GLUTs and SREBP2

After treating HEB cells with metformin, simvastatin, and their combination, there was a significant decrease in both the mRNA and protein levels of GLUT1 and GLUT6 (Fig. 4e, f). Conversely, the mRNA and protein levels of SREBP2 precursor increased, while the mature form of SREBP2 decreased (Fig. 4e, f). For the U87 and T98G cell lines, there was a significant decrease in both the mRNA and protein levels of GLUT1/6. Conversely, the mRNA and protein levels of SREBP2 precursor increased, while the mature form of SREBP2 decreased. In U87 cells, the use of metformin alone resulted in the most noticeable increase in SREBF2 (Fig. 4g–j).

### The Combined Use of Metformin and Simvastatin Inhibits SREBP2 Maturation

**Fig. 5:**
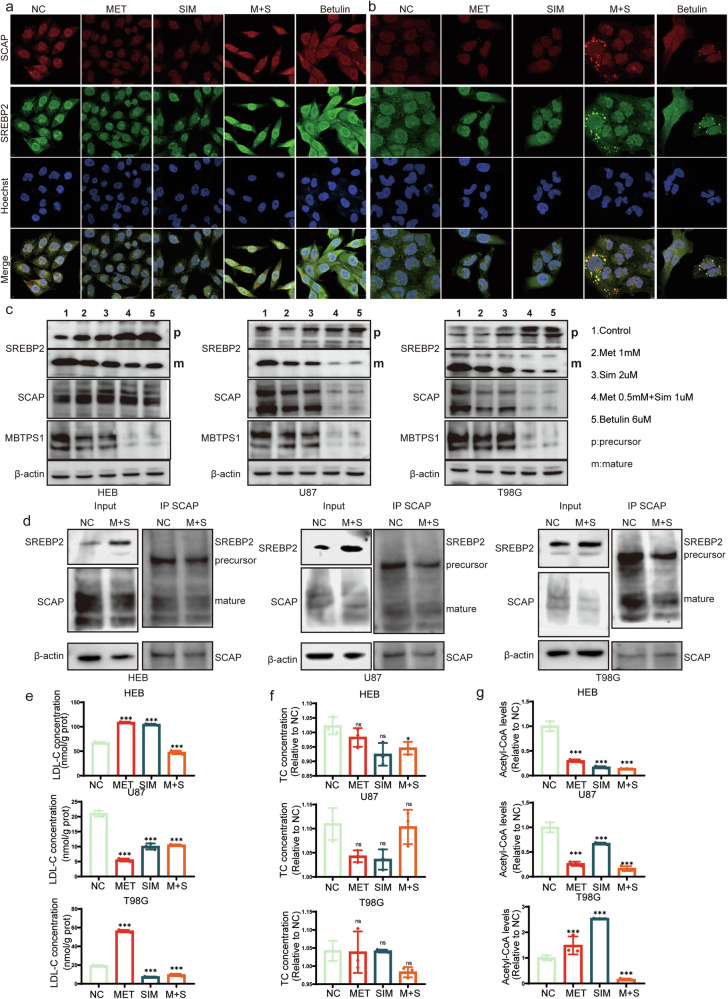
Inhibition of SREBP2 Maturation and Increase in Precursor Form by Combined Metformin and Simvastatin Treatment. **a**, **b** Immunofluorescence was staining to assess alterations in the co-localization of SCAP and SREBP2 following treatment with Metformin, Simvastatin and Betulin in HEB (**a**) and T98G (**b**) cells. **c** Alterations in the levels of SREBP2 precursor and mature forms, along with SCAP and MBTPS1 proteins, in HEB, U87, and T98G cells following treatment with Metformin, Simvastatin and Betulin. **d** Immunoprecipitation (IP) assay was conducted to explore alterations in SREBP2 precursor and mature forms under consistent SCAP levels after the concurrent administration of Metformin and Simvastatin. **e** Changes in LDL-C levels following Metformin and Simvastatin treatment. **f** Changes in TC levels following Metformin and Simvastatin treatment. **g** Changes in Acetyl-CoA levels. Metformin was used at a concentration of 1 mM, simvastatin at 2 µM, and in combination, and the concentrations were 0.5 mM for metformin and 1 µM for simvastatin. Additionally, betulin was used at a concentration of 6 µM. Statistical significance is denoted by *P < 0.05 and ***P < 0.001, as determined by a two-tailed Student’s t-test.

In HEB cell line, the individual use of metformin, simvastatin, or their combination did not induce significant changes in the localization of SCAP and SREBP2. Similarly, in T98G and U87 cells, the individual use of metformin or simvastatin did not alter the localization of SCAP and SREBP2. However, the combined use of metformin and simvastatin led to the accumulation of SREBP2 and SCAP in vesicles, preventing their entry into the cell nucleus. Betulin, used as a positive control, has a similar effect on inhibiting SREBP2 maturation as the combined treatment of metformin and simvastatin (Fig. 5a, b and Fig. S1d).

After drug treatment, there was an increase in the SREBP2 precursor in HEB cells, with the mature form showing the greatest reduction upon the combined use of both drugs, while SCAP did not exhibit significant changes. MBTPS1 (Membrane-bound transcription factor protease site 1) is a protease that functions on the endoplasmic reticulum membrane [29]. One of its primary functions is involvement in the activation of SREBP. The expression of MBTPS1 was downregulated. In U87 and T98G cells treated with metformin and simvastatin, there was a significant downregulation in the expression of the mature form of SREBP2, MBTPS1, and SCAP. The inhibitory effect of betulin on SREBP2 maturation in HEB cells is weaker than in U87 and T98G cells, showing a pattern similar to that of the combined treatment with metformin and simvastatin (Fig. 5c). Additionally, using SCAP as a reference, we compared the levels of SREBP2 precursor and mature forms with or without drug treatment. We found that after combined drug treatment, the mature form of SREBP2 decreased while the precursor increased (Fig. 5d).

### The Impact of Metformin and Simvastatin on Lipid Metabolism

After treating HEB, U87, and T98G cells with metformin and simvastatin, the intracellular Low-Density Lipoprotein Cholesterol (LDL-C) significantly decreased following the combined treatment (Fig. 5e). Moreover, the Total Cholesterol (TC) content showed no significant changes within the same samples, indicating that metformin and simvastatin do not affect cholesterol uptake (Fig. 5f). Regarding the material for cholesterol synthesis, the levels of Acetyl-CoA are decreased whether metformin is used alone, simvastatin is used alone, or both are used in combination (Fig. 5g).

### Mitochondrial Membrane Potential and Alkaline Comet Assay

**Fig. 6:**
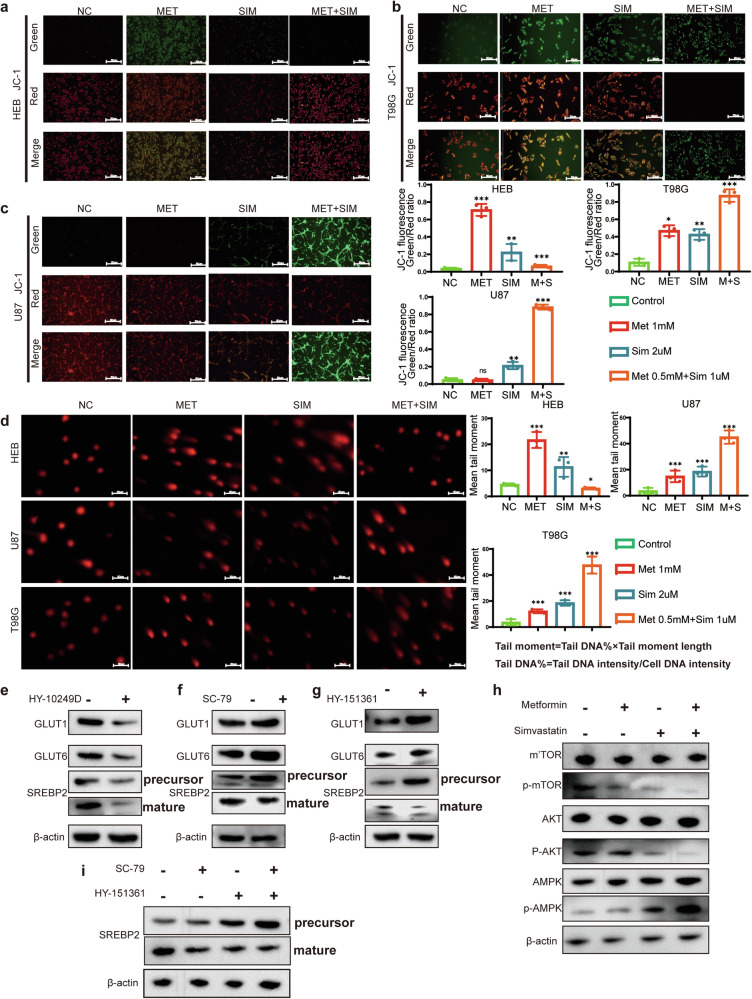
Identification of Mitochondrial Potential and DNA Damage in Cells Induced by Metformin and Simvastatin. **a**–**c** JC-1 staining assay indicated the enrichment of J-monomers in HEB, U87, and T98G cells after treatment with a combination of Metformin and Simvastatin. In untreated HEB, U87, and T98G cells, J-aggregates were enriched. **d** In the comet assay, tail lengths of HEB, U87, and T98G cells treated with a combination of Metformin and Simvastatin were significantly prolonged compared to the negative control, while tail length was not prolonged in untreated HEB, U87, and T98G cells. **e**–**g** Western blot analysis of T98G cells treated with AKT inhibitor (HY-10249D), AKT activator (SC-79) and AMPK inhibitor (HY-151361) revealed alterations in GLUT1, GLUT6, and SREBP2 protein levels; **h** Metformin and Simvastatin combination antagonizes the AKT pathway and activates the AMPK pathway; **i** Changes in the precursor and mature forms of SREBP2 in T98G cells after treatment with AKT activator (SC-79) and AMPK inhibitor (HY-151361). Metformin was used at a concentration of 1 mM, simvastatin at 2 µM, and in combination, and the concentrations were 0.5 mM for metformin and 1 µM for simvastatin. All experiments were conducted in triplicate, and the data are expressed as the mean ± standard deviation. Statistical significance is denoted by *P < 0.05, **P < 0.01, and ***P < 0.001, as determined by a two-tailed Student’s t-test.

Mitochondrial Membrane Potential (MMP) refers to the electrical potential difference between the inner and outer membranes of a cell’s mitochondria [30]. Within the cell, the normal maintenance of mitochondrial membrane potential is crucial for the cell’s survival and function. A decrease in mitochondrial membrane potential may indicate apoptosis or other cellular damage processes [31]. In this experiment, we used metformin, simvastatin, and their combination. We tested the mitochondrial membrane potential in HEB, U87, and T98G cells. We found that normal astrocytes (HEB) showed higher tolerance to the combination treatment of metformin and simvastatin compared to glioma cells (U87, T98G) (Fig. 6a–c). However, it’s worth noting that high doses of metformin alone caused significant changes in the membrane potential of HEB cells (Fig. 6a). In the case of T98G cells, regardless of whether metformin or simvastatin was used alone or in combination, a significant decrease in membrane potential was observed, consistent with the increase in apoptosis induced by metformin and simvastatin as mentioned earlier **(**Fig. 6b**)**. Considering that the use of metformin and simvastatin led to increased apoptosis and decreased mitochondrial membrane potential in glioma cell lines, we speculate whether these two drugs cause DNA damage in glioma cells. HEB exhibit higher tolerance to the combination therapy, as indicated by smaller tail moment values. However, they are intolerant to high doses of metformin when used alone. In contrast, glioma cell lines U87 and T98G show extreme intolerance to the combination therapy, evident from the significantly larger tail moment values, indicating substantial DNA damage caused by the combined use of metformin and simvastatin. Additionally, these glioma cells exhibit DNA damage when treated with metformin or simvastatin alone (Fig. 6d).

### Metformin and simvastatin play a crucial role in regulating cellular metabolic pathways

Previous studies have reported that Metformin can regulate the AMPK pathway [32] and cell cycle [33], while simvastatin can modulate the mTOR [34] and SREBP pathways [35]. The main focus of this study is to investigate whether metformin and simvastatin synergistically target GLUTs and SREBP2, exploring the combined effects of these drugs on glioma metabolism, proliferation, and apoptosis. In U87 cells, treatment with AKT inhibitor (HY-10249D) resulted in downregulation of GLUT1 and GLUT6 expression, while SREBP2 expression was downregulated (Fig. 6e). Conversely, treatment with AKT activator (SC-79) led to upregulation of GLUT1/6 expression, an increase in the precursor form of SREBP2, and no significant change in the mature form of SREBP2 (Fig. 6f).Treatment with AMPK inhibitor (HY-151361) led to upregulation of GLUT1 and GLUT6 expression and downregulation of SREBP2-n expression (Fig. 6g). Using metformin alone can activate the AMPK pathway and inhibit the AKT pathway, while using simvastatin alone only inhibits the AKT pathway. However, the combined use of both drugs shows a more significant effect in inhibiting AKT and activating AMPK (Fig. 6h). Using an AKT activator (SC-79) and an AMPK inhibitor (HY-151361) to simulate the over activation of tumor pathways, we observed a significant increase in the precursor and active forms of SREBP2 (Fig. 6i).

### Metformin and simvastatin regulate energy metabolism

**Fig. 7:**
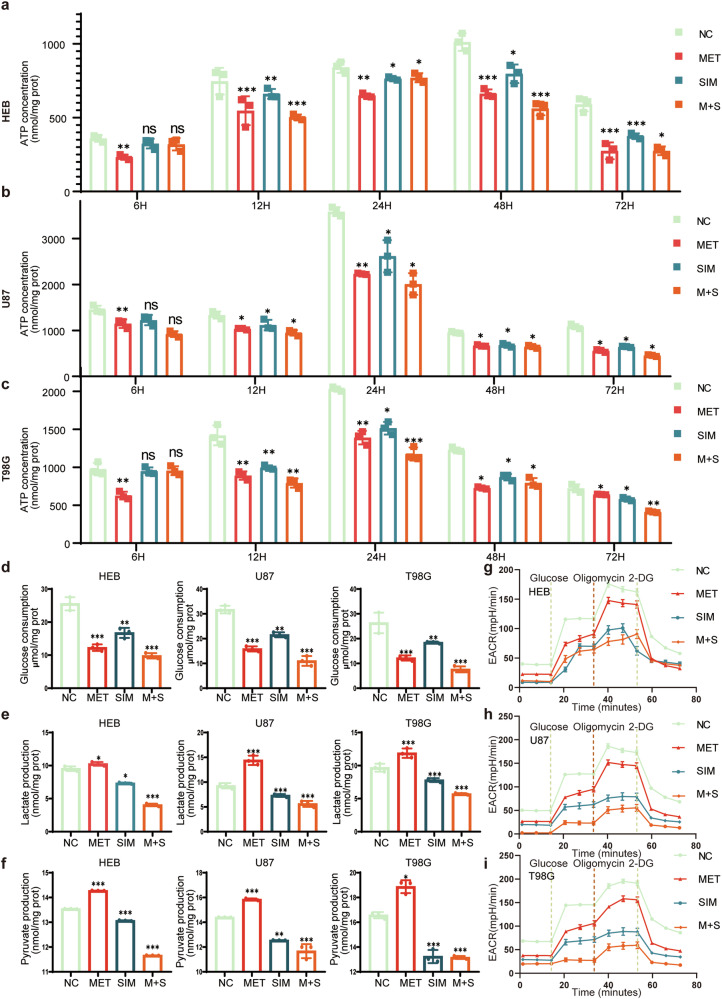
Changes in cellular energy and glucose metabolism products after treatment with Metformin and Simvastatin. **a**–**c** Changes in cellular ATP levels after treatment with metformin and simvastatin in HEB, U87, and T98G cells at 6 h, 12 h, 24 h, 48 h, and 72 h; **d** Glucose uptake in HEB, U87, and T98G cells after 48 h of treatment with metformin and simvastatin; **e** Lactic acid levels in HEB, U87, and T98G cells after 48 h of treatment with metformin and simvastatin; **f** pyruvate levels in HEB, U87, and T98G cells after 48 h of treatment with metformin and simvastatin. All experiments were conducted in triplicate, and the data are expressed as the mean ± standard deviation. **g**–**i** Glucose, oligomycin, and 2-DG were measured in HEB, U87, and T98G cells after 48 h of treatment with metformin and simvastatin in Seahorse analysis. Statistical significance is denoted by *P < 0.05, **P < 0.01, and ***P < 0.001, as determined by a two-tailed Student’s t-test. All experiments were conducted in triplicate, and the data are expressed as the mean ± standard deviation. Metformin was used at a concentration of 1 mM, simvastatin at 2 µM, and in combination, and the concentrations were 0.5 mM for metformin and 1 µM for simvastatin. Statistical significance is denoted by *P < 0.05, **P < 0.01, and ***P < 0.001, as determined by a two-tailed Student’s t-test.

Within the cell, the normal maintenance of mitochondrial membrane potential is crucial for the cell’s survival and function. A decrease in mitochondrial membrane potential may indicate apoptosis or other cellular damage processes. After drug treatment, regardless of whether metformin, simvastatin, or their combination was used, the ATP levels in HEB cells showed a general downward trend. At 6 h after treatment, the decrease in ATP levels was not significant. Subsequently, at 12 h and 24 h, ATP production was maintained at relatively high concentrations. However, after 24 h, the ATP production in HEB cells gradually decreased under the influence of metformin and simvastatin, showing little difference from the control group. Furthermore, the baseline ATP levels in HEB cells remained relatively high throughout the experiment (Fig. 7a). For the U87 cell line, during the initial 12 h of treatment, there was not a significant change in ATP levels. Starting from 24 h, the effects of metformin and simvastatin became apparent, consistently maintaining ATP levels at a lower level. Moreover, compared to using metformin or simvastatin alone, the combination of both drugs had a significant impact (Fig. 7b). For the T98G cell line, the overall trend was similar to the U87 cells. After drug treatment, ATP production showed a general decrease, and this trend became more significant over time (Fig. 7c).

After drug treatment, HEB, U87, and T98G cells all exhibited reduced glucose uptake. Among them, the combined use of both drugs had the most significant impact on glucose uptake (Fig. 7d). After treatment with metformin alone, lactate levels increased abnormally in HEB, U87, and T98G cells, possibly related to the side effects of metformin. However, when simvastatin was used alone or in combination, lactate levels were reduced in these cells (Fig. 7e). The trend in acetate levels verified a pattern similar to lactate. The combination of both drugs significantly reduced the production of acetate (Fig. 7f).

In the Seahorse analysis, the HEB cell line exhibited significant changes in energy metabolism after drug treatment compared to untreated cells, with the combined use of metformin and simvastatin showing no advantage over individual drug treatments. For the tumor cell lines U87 and T98G, both the combined and individual drug treatments significantly inhibited energy metabolism, with the combined treatment showing the strongest inhibitory effect (Fig. 7g–i).

### Exploring the In Vivo Effects of Metformin and Simvastatin in Regulating the Malignant Progression of Glioblastoma

**Fig. 8:**
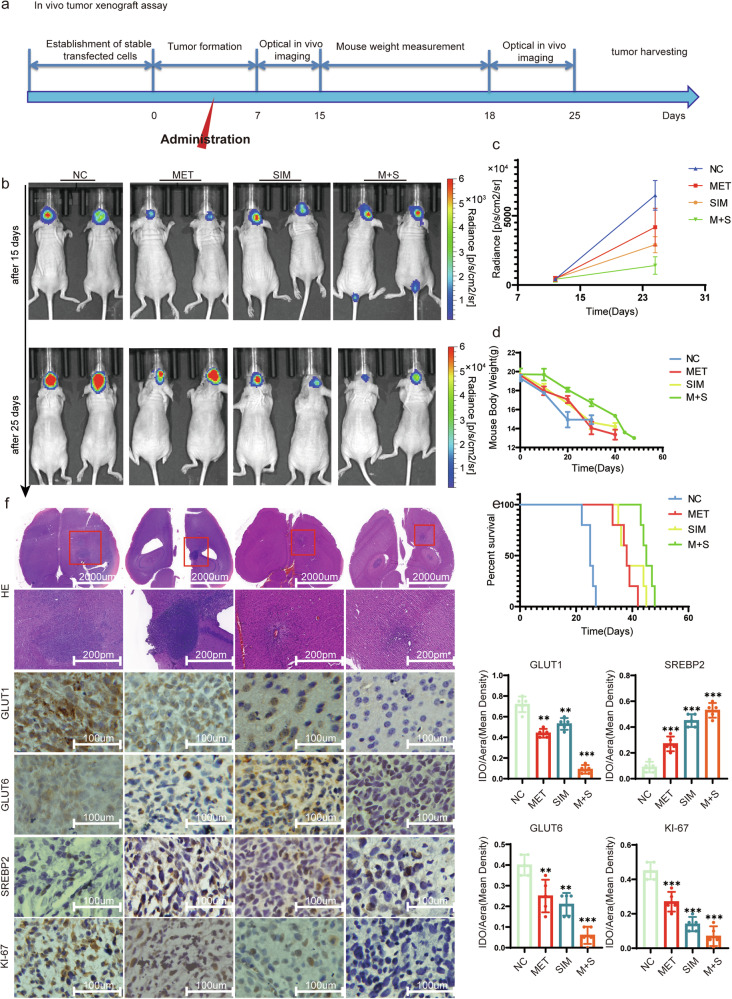
In vivo verification of the tumor-suppressive impact of both metformin and simvastatin. **a** The timeline illustrates the progression of intracranial xenograft tumors of U87-luc in response to metformin and simvastatin, with different stages indicated by distinct arrows. **b** Bioluminescence imaging of xenograft tumors in mice was shown, Fluorescence imaging was conducted on the 15th and 25th days after xenograft tumor transplantation, respectively. **c** Statistics of tumor fluorescence intensity in mice. **d** Mouse body weight was measured, with the first measurement taken at the initial tumor imaging and subsequent measurements conducted every three days thereafter. **e** The survival of mice was measured. **f** Mouse intracranial tumor H&E staining and IHC for the expression of GLUT1, GLUT6, SREBP2, and Ki-67 in the tumor. All experiments were conducted in triplicate, and the data are expressed as the mean ± standard deviation. Statistical significance is denoted by **P < 0.01, and ***P < 0.001, as determined by a two-tailed Student’s t-test.

After treating mice with metformin and simvastatin according to the standard intracranial xenograft procedure, the tumor volume significantly reduced compared to the untreated control group (Fig. 8a, b). We measured the fluorescence intensity in treated mice and found that the combination therapy of metformin and simvastatin significantly reduced tumor fluorescence intensity compared to the individual use of metformin and simvastatin (Fig. 8c). Moreover, the combination therapy showed the most significant extension in the survival period of the mice and had the least impact on the mice’s body weight (Fig. 8d, e). After performing H&E staining and immunohistochemistry on mouse brain tumor sections, it was observed that the tumor volume in the treated group significantly decreased. Additionally, the expressions of GLUT1, GLUT6, and Ki-67 were downregulated, while SREBP2 expression increased in the treated group (Fig. 8f).

## Discussion

In this study, we conducted a comprehensive exploration of the combined effects of metformin and simvastatin on inhibiting glioma progression. Our focus was on understanding their impact on GLUTs and SREBP2 expression. We also delved into the influence of these drugs on the mTOR and AMPK pathways, aiming to uncover their implications for glioma cell metabolism, proliferation, and apoptosis. Additionally, we validated our findings through in vivo experiments in an animal model. The results presented here shed light on the potential of combined metformin and simvastatin therapy as a novel approach for glioma treatment, addressing several key aspects.

Our findings support previous research indicating that combining metformin and simvastatin enables a reduction in individual drug dosages, enhancing the safety profile of the treatment regimen. When used alone, metformin can induce apoptosis in glioma cells, but at significantly high concentrations [36, 37]. However, when combined with simvastatin, the required metformin dosage is reduced by at least half, while simultaneously enhancing its inhibitory effects on glioma cell proliferation. Tumor cells often acquire the necessary energy through high glucose intake and lactic acid fermentation (the Warburg effect) [38, 39]. However, the combined use of metformin and simvastatin may alter this metabolic pattern. Metformin [40] is typically used to lower blood sugar, while simvastatin [41] is used to lower cholesterol. The combination of these two drugs may suppress the tumor cells’ dependency on glucose and also impact cholesterol metabolism.

Low-grade gliomas exhibit higher levels of SREBP2 expression compared to high-grade gliomas (Fig S1c). However, the expression levels of SREBP2 in both types of gliomas are lower than those in normal brain tissue. This suggests abnormalities in cholesterol synthesis and metabolic pathways regulated by SREBP2 in tumor cells. Normal cells typically regulate cholesterol levels through SREBP2 [42], but tumor cells may have lost this self-regulation mechanism. Our previous research indicated that overexpression of SREBP2 leads to increased expression of GLUT1/6 [6]. Previous studies have clarified the effects of metformin on the GLUTs family [43] and simvastatin [44] on the SREBP2 family. However, in our study, the combined use of these drugs restored SREBP2 expression but significantly reduced the expression levels of GLUT1/6. Although the combined use of metformin and simvastatin alters energy metabolism, leading to an increase in SREBP2 levels, the elevated SREBP2 precursor is likely to stem from disrupted lipid metabolism. Additionally, the mature form of SREBP2, acting as a transcription factor, exhibits decreased expression levels after drug treatment.

In glioma cells, the action of metformin involves activating AMPK, inhibiting cell growth and metabolism, thereby suppressing tumor development [32, 45, 46]. Simvastatin, on the other hand, slows down the proliferation rate of glioma cells by inhibiting the AKT pathway, thus inhibiting tumor growth [47, 48]. Metformin alone is insufficient to inhibit the AKT pathway, while simvastatin alone has difficulty activating the AMPK pathway. Remarkably, the combination therapy efficiently activates the AMPK pathway while suppressing the mTOR pathway. This dual modulation is crucial for altering glioma cell energy metabolism, inhibiting proliferation, and inducing apoptosis. Moreover, the combination treatment significantly reduces glucose absorption and lactate production, countering the Warburg effect and impeding the glycolytic shift associated with the Warburg effect.

In animal models, the combination of metformin and simvastatin led to a significant reduction in tumor volume and prolonged the survival of mice, affirming the effectiveness of this approach in vivo. Immunohistochemical analysis further substantiated these results, demonstrating decreased expressions of GLUT1, GLUT6, and Ki-67, accompanied by increased SREBP2 expression. These findings underscore the clinical relevance of the combination therapy and its potential as a promising treatment strategy for gliomas.

## Conclusion

In conclusion, this study provides compelling evidence supporting the synergistic effects of metformin and simvastatin in inhibiting glioma progression. By combining metformin and simvastatin, the transition of SREBP2 precursor to its mature form is inhibited, resulting in a downregulation of SREBP2 mature form, making it challenging to act as a transcription factor to alter GLUT1/6 levels. Furthermore, the combined use of these two drugs significantly reduces the glycolipid metabolism levels in tumor cells, including ATP, lactate, acetoacetate, and LDL-C, reshaping the metabolic profile of glioma. This offers a new approach for the diagnosis and treatment of glioma. The ability to enhance therapeutic efficacy while minimizing drug dosage and adverse effects makes this strategy particularly promising for further exploration in clinical settings. Nonetheless, further investigations are warranted to fully elucidate the underlying mechanisms and optimize the treatment protocol for maximal clinical benefit.

## Supplementary information

Supplementary Materials

original data files

## Data Availability

The datasets used and/or analyzed during the current study are available from the corresponding author on reasonable request.
