# Supplementary material for: Combined metformin and simvastatin therapy inhibits SREBP2 maturation and alters energy metabolism in glioma
Source: Cell Death Dis. 2024 Nov 9;15(11):809. doi: 10.1038/s41419-024-07169-5 (PMC11550444; doi:10.1038/s41419-024-07169-5)
Supplement: Supplementary file 1 — Supplementary Materials [file 41419_2024_7169_MOESM1_ESM.pdf]

Supplementary Materials and Methods

Data Processing and Differential Expression Analysis

In this study, data processing and differential expression analysis were conducted using the Gene Expression Profiling Interactive Analysis (GEPIA) online database (http://gepia2.cancer-pku.cn/). GEPIA is a valuable online cancer microarray database utilized for exploring expression differences and prognostic correlations.

Gene expression correlation analysis was carried out using specific sets of The Cancer Genome Atlas (TCGA) expression data. The Spearman method was employed to determine the correlation coefficients. In the generated plots, LAYN was plotted on the x-axis, while other genes of interest were represented on the y-axis. Both tumor and normal tissue datasets were utilized for the analysis.

Immunohistochemistry

In this study, tissue samples from different grades of human gliomas (WHO grade I-IV) as well as normal brain tissues were formalin-fixed and paraffin-embedded to create 4 mm sections. To facilitate antigen retrieval, these sections were boiled in sodium citrate buffer (pH 6.0). To block endogenous horseradish peroxidase (HRP) activity, 3% H2O2 was applied. The slides were then treated with a 10% normal goat serum and incubated with primary antibodies at 4°C overnight.

The detection of signals was achieved using a secondary antibody conjugated with horseradish peroxidase and 3, 3'-diaminobenzidine (DAB) as the substrate. Subsequently, the slides were counterstained with hematoxylin. Representative images were captured using an Olympus inverted microscope. The staining intensity of glioma samples was quantified using the H-score method.

Immunofluorescence assays

Cells were grown on glass coverslip in confocal dish and were treated accordingly. Subsequently, the cells were fixed with 4% paraformaldehyde in PBS for 15 min, and 0.5% of Triton solution was added to penetrate the cytomembrane. After 12h incubation with primary antibodies at 4 °C, DAPI and fluorescence-dye conjugated staining kit secondary antibodies were used to detect the binding of primary antibody and the nuclei of fixed cells. Visualization of fixed cells was carried out using a confocal laser scanning microscope (Leica, STELLARIS 5 Cryo).

RNA Extraction and Real-time Polymerase Chain Reaction (PCR)

For RNA extraction, TRIzol reagents (Thermo, MA, USA) were employed following the manufacturer's guidelines. Subsequently, complementary DNA (cDNA) was synthesized using the GoScript reverse transcription system (TransGen Biotech, AE341-02).

Quantitative real-time PCR was conducted using TransStart Green qPCR SuperMix (TransGen Biotech, AQ111-02) on an ABI7500 real-time PCR system (Applied Biosystems), adhering to standard procedures. To normalize the cycle threshold (Ct) values for each gene, GAPDH expression levels were used as the reference. The $2^{-\Delta\Delta Ct}$ method was applied for quantitative analysis, facilitating accurate comparison of gene expression levels across different samples.

The specific primers used in this study can be found in Table S2, ensuring the specificity and reliability of the PCR reactions for the targeted genes.

Western blot assay

For western blot analysis, samples were separated on SDS-PAGE gels and transferred to PVDF membranes. Membranes were processed following the ECL western blot protocol by GE Healthcare. The following antibodies were used: anti-SREBP2 (1:500, Abcam, ab30682), anti-GLUT1 (1:500, Santa Cruz, sc-377228), anti-GLUT4 (1:500, Santa Cruz, sc-53566), anti-GLUT6 (1:500, Santa Cruz, sc-373973), anti-mTOR (1:1000, Santa Cruz, sc-517464), anti-p-mTOR (1:1000, Santa Cruz, sc-293133), anti-AKT1 (1:1000, Santa Cruz, sc-5298), anti-pAKT1 (1:1000, Santa Cruz, sc-135650), anti-AMPK α1 (1:1000, CST, 2532), anti-p-AMPKα (1:1000, CST, 2535), anti-SCAP (1:1000, abcam, ab190103), anti-MBTPS1 (1:1000, Thermo, PA5-77103), and β-actin (1:1000, Sangon, D110001). Antibody validation was confirmed through the manufacturers' websites.

Cell Transfection

Small interfering RNAs (siRNAs) targeting GLUT1 and GLUT6, along with a negative control (NC) siRNA, were obtained from Thermo. Cell transfections were conducted using Lipofectamine® 2000 reagent (Thermo Fisher Scientific, Inc.) at 37 °C, with a concentration of 10 nM for vectors and 40 nM for siRNAs. The cells were incubated with the transfection mixtures for 24 h.

To assess transfection efficiency, both reverse transcription quantitative polymerase chain reaction (RT-qPCR) and western blot assays were performed. The primers used for RT-qPCR are provided in Table S2. These procedures ensured effective modulation of GLUT1 and GLUT6 expression in the cells for subsequent analysis.

Cell Invasion and Migration Assays

The migration and invasion capabilities of T98G and U87 cells were evaluated using Transwell chambers with 8 μm pore size and 24-well inserts (Costar). For the invasion assay, the insert chamber membrane was coated with diluted matrigel (BD Biosciences) mixed with serum-free medium (1:40). After 48 hours of transfection, cells (0.8×10^4) in serum-free medium were added to the upper chamber, while the lower chamber contained medium with 10% fetal bovine serum (FBS).

In the migration assay, the same procedure was followed, except matrigel was not used.

Following incubation, cells that migrated or invaded into the lower chambers were counted under an inverted microscope (Olympus). These assays provided valuable insights into the cells' ability to migrate and invade crucial factors in cancer metastasis.

Cell Proliferation Assay

Following a 48-hour period of transfection with Metformin or Simvastatin, T98G , U87,LN229,U251 and HEB cells in the exponential growth phase were seeded into 96-well plates at a density of 4×10^3 cells per well. Cell proliferation was assessed using the cell-counting kit-8 (CCK-8, TransGen) according to the manufacturer's instructions. This assay allowed for a quantitative analysis of cell viability and proliferation in response to the treatments, providing essential information about the effects of Metformin and Simvastatin on cell growth.

TUNEL Assay

To determine the apoptotic rate, T98G, U87, and HEB cells in the exponential growth phase were seeded into 96-well plates at a density of 4×10^3 cells per well after 48 hours of transfection. The cells were fixed in a 4% formaldehyde solution for 20 minutes at room temperature and washed three times with PBS (Beyotime, Wuhan, China).

The TUNEL assay kit (Beyotime, Shanghai, China) containing terminal deoxynucleotidyl transferase (TdT) was prepared immediately before use following the manufacturer's protocol. Apoptotic cells in the sections were detected using a microscope (Nikon Corp., Tokyo, Japan). This assay allowed for the identification and quantification of apoptotic cells, providing crucial insights into the cellular response to the experimental conditions.

IP Assay

U87-MG cells, T98G-MG cells and HEB cells were collected and incubated with the protein-A/G MagBeads (beyotime, Shanghai, China) and antibody. After overnight incubation, the immune

complexes were centrifuged and washed, and the proteins were detected by western blot with an anti-SCAP antibody (Santa, USA) and anti-SREBP2 antibody (ABclonal, China) according to the manufacturer's protocol.

Determination of Intracellular Acetyl-CoA Levels Using ELISA Assay

To evaluate the influence of metformin and simvastatin on acetyl coenzyme A (Ac-CoA) (sangong, China)levels in U87, T98G, and HEB cells, we collected treated cells for each of the three cell lines and meticulously prepared standard curves in accordance with the manufacturer's guidelines. Following the established protocols, standard curves were meticulously generated. Subsequently, the optical density (OD) values of both the treatment and control groups were quantified using an enzyme-linked immunosorbent assay (ELISA) reader, with measurements taken at a wavelength of 450nm. Through a thorough analysis of these data, we aim to discern the extent of each drug's impact on Ac-CoA levels within distinct cell lines.

Measuring Total Cholesterol (TC) Content and Low-Density Lipoprotein (LDL) Content involved the utilization of specialized assay kits. The TC level was determined utilizing the TC Assay Kit from Solaibao, China, in adherence to the manufacturer’s protocol. Evaluation of LDL Content was conducted using the Lactic Acid (LDL) HPLC Assay Kit from Solaibao, China, following the provided instructions. These assays are crucial for investigating cellular energy metabolism and diverse metabolic processes.

The Dual-Luciferase reporter gene assay

The Dual Luciferase Reporter Gene Assay Kit, purchased from Yeasen Biotechnology (Shanghai) Co., Ltd. with catalog number 11402ES60. All procedures should be carried out according to the instruction manual. The constructed site information is as follows.

Table S1 Samples information represented in Figure4b, Figure5b, h, Figure s3a, Figure s5a

Variable		Samples (n=102)
Sex		
	Male	53
	Female	49
Age(year)		
	≤50	42
	>50	60
WHO grade		
	Ⅰ	2
	Ⅱ	19
	Ⅲ	16
	Ⅳ	65
Location		
	Frontal	41
	Parietal	8
	Occipital	11
	Temporal	42
Histology		
	Oligodendroglioma	16
	Astrocytome	13
	Diffuse midline glioma	17
	Glioblastoma	56

TableS2 Oligos used in the study

SREBF2-F	CTGGGAGACATCGACGAGAT
SREBF2-R	GACCTGGGTGAATGACCGTT
SLC2A1-F	CTTCACTGTCGTGTCGCTGT
SLC2A1-R	GGCCACGATGCTCAGATAGG
SLC2A2-F	CAATGCACCTCAACAGGTAATAA
SLC2A2-R	TGGCTTTGATTCTTCCAAGTGT
SLC2A4-F	ACCTTGGTCTCGGTGTTGTT
SLC2A4-R	GGCCACGATGAACCAAGGAA
SLC2A6-F	AACGTCCGGAGACAGAGCA
SLC2A6-R	GGTGCTGTCGAAGATGGACT
GAPDH-F	TGACTTCAACAGCGACACCCA
GAPDH-R	CACCCTGTTGCTGTAGCCAAA

Table S3.1: Half-maximal inhibitory concentrations (IC50) of different drugs in cell lines after 48 hours of treatment

	HEB	U87	T98G	LN229	U251
Metformin (mM)	9.516	6.945	7.204	2.885	9.046
Simvastatin (uM)	37.95	13.98	11.78	2.320	15.67

Table S3.2: Concentrations of drugs used in combination therapy

	HEB	U87	T98G	LN229	U251
Metformin (mM)	2.5	1.5	1.5	0.5	2.5
Simvastatin (uM)	5.0	2.5	2.5	0.5	4.0

Table S4: The binding sites of SREBP2 with SLC2A1/6

Gene	Fragment length	Binding site		Fluorescence intensity (Luc value)		
		name	sequence	control group	experimental group	ratio
SLC2A1	-257bp～+16bp	836	AGGGGGTGAG	56949.8	98279.0	1.725
	-737bp～-257bp	764	AGGAGGTGAT	59387.2	87817.8	1.478
	-764bp～-737bp	737	CACCTGAACT	56045.6	176544.8	3.150
	-836bp～-764bp	257	CTGACATGAT	54900.0	223402.5	4.069
SLC2A6	-263bp～+7bp	833	GGGGCGTGAC	60216.6	172167.8	2.859
	-357bp～-263bp	357	CAGGCGTGAC	58292.75	297511.0	5.104
	-833bp～-357bp	263	AGGGCGTGAT	63191.0	187811.0	2.972

Supplemental legend

Fig.S1 Supplemental Experimental Data

(a) Kaplan-Meier plots of overall survival in glioma patients with high and low expressions of GLUT1, GLUT6, GLUT4, SREBP1, SREBP2 and VEGF; (b) Bioinformatics analysis indicated that GLUT1, GLUT6, GLUT4, SREBP1, SREBP2 and VEGF expression level in GBM tissues (n = 163)was significantly upregulated than normal tissues(n = 207); (c) SREBP2 mRNA expression in gliomas is generally lower than in normal brain tissue, and the expression level of SREBP2 is lower in high-grade gliomas compared to low-grade gliomas; (d) Immunofluorescence staining to detect the localization of SCAP and SREBP2 in U87 cells.

Fig.S2 Measurement of Half Inhibitory Concentration (IC50) for Metformin and Simvastatin in Cells

(a) U87, LN229, T98G, U251, and HEB cells were treated with Metformin, and cell proliferation was assessed using CCK8 assay after 48 hours (n=3); (b) IC50 values were calculated using GraphPad Prism 7. (c) U87, LN229, T98G, U251, and HEB cells were treated with Simvastatin, and cell proliferation was assessed after 48 hours (n=3); (d) IC50 values were calculated.(e) After 48 hours of treatment with low concentrations of metformin, simvastatin, and their combination, cell viability decreased significantly. All experiments were conducted in triplicate, and the data are expressed as the mean ± standard deviation. Statistical significance is denoted by *$P < 0.05$, **$P < 0.01$, and ***$P < 0.001$, as determined by a two-tailed Student's t-test.

Fig.S3 Primary Glioma SREBP2 Expression

Supplemental immunohistochemistry images illustrate the expression levels of SREBP2 precursor and mature forms in primary gliomas of different grades.

Fig.S4 Primary and Recurrent Glioma SREBP2 Expression

Supplemental immunohistochemistry images depict the expression levels of SREBP2 precursor and mature forms in primary and recurrent gliomas of varying grades.

Figure S1

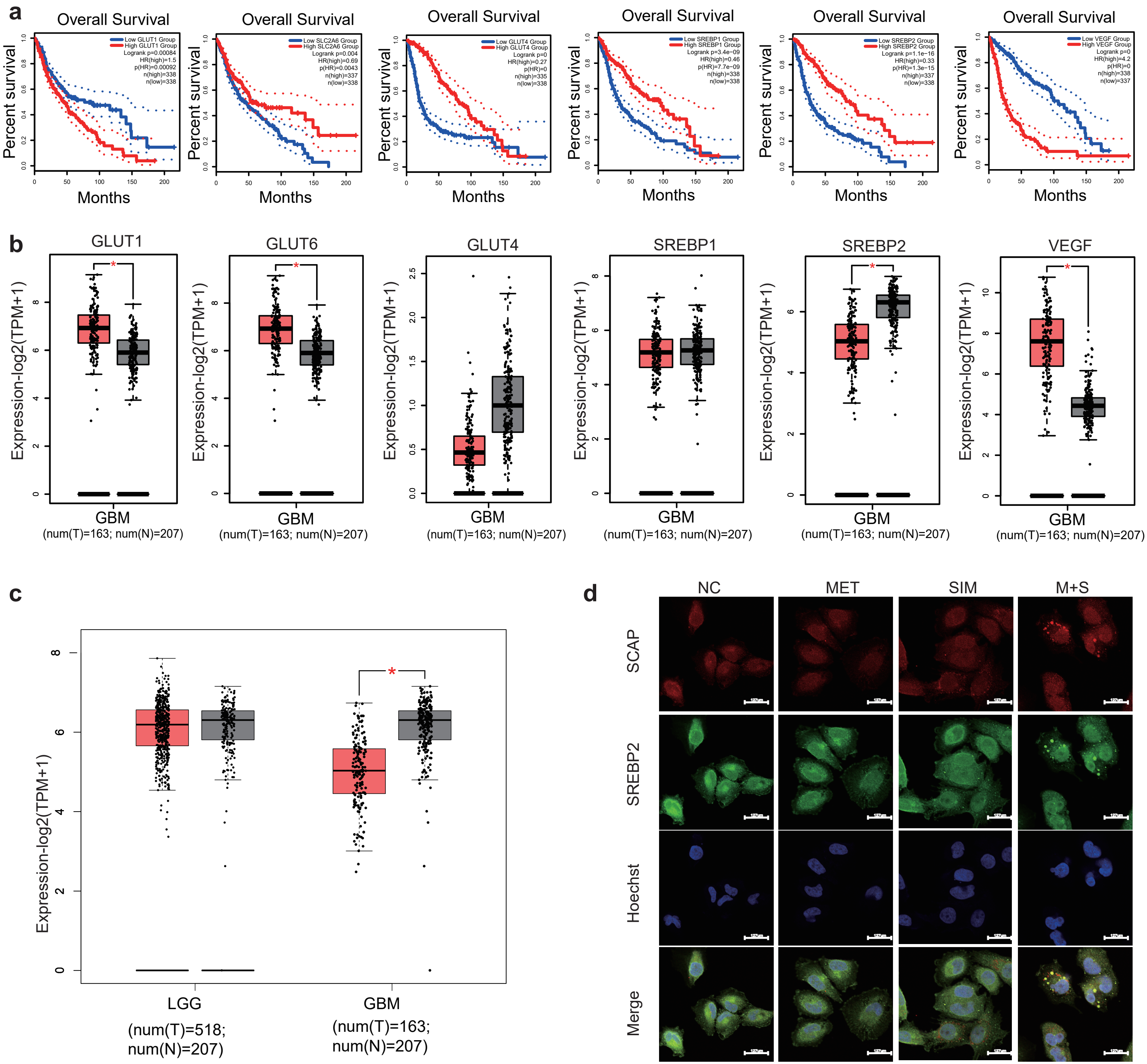

a
Overall Survival
Percent survival
Months
Low GLUT1 Group
High GLUT1 Group
Logrank p=0.00084
HR(high)=1.5
p(HR)=0.00092
n(high)=338
n(low)=338
Low SLC2A6 Group
High SLC2A6 Group
Logrank p=0.004
HR(high)=0.69
p(HR)=0.0043
n(high)=337
n(low)=338
Low GLUT4 Group
High GLUT4 Group
Logrank p=0
HR(high)=0.27
p(HR)=0
n(high)=335
n(low)=338
Low SREBP1 Group
High SREBP1 Group
Logrank p=3.4e-09
HR(high)=0.46
p(HR)=7.7e-09
n(high)=338
n(low)=338
Low SREBP2 Group
High SREBP2 Group
Logrank p=1.1e-16
HR(high)=0.33
p(HR)=1.3e-15
n(high)=337
n(low)=338
Low VEGF Group
High VEGF Group
Logrank p=0
HR(high)=4.2
p(HR)=0
n(high)=338
n(low)=337
b
GLUT1
GLUT6
GLUT4
SREBP1
SREBP2
VEGF
Expression-log2(TPM+1)
GBM
(num(T)=163; num(N)=207)
c
Expression-log2(TPM+1)
LGG
(num(T)=518; num(N)=207)
GBM
(num(T)=163; num(N)=207)
d
NC
MET
SIM
M+S
SCAP
SREBP2
Hoechst
Merge

Figure S2

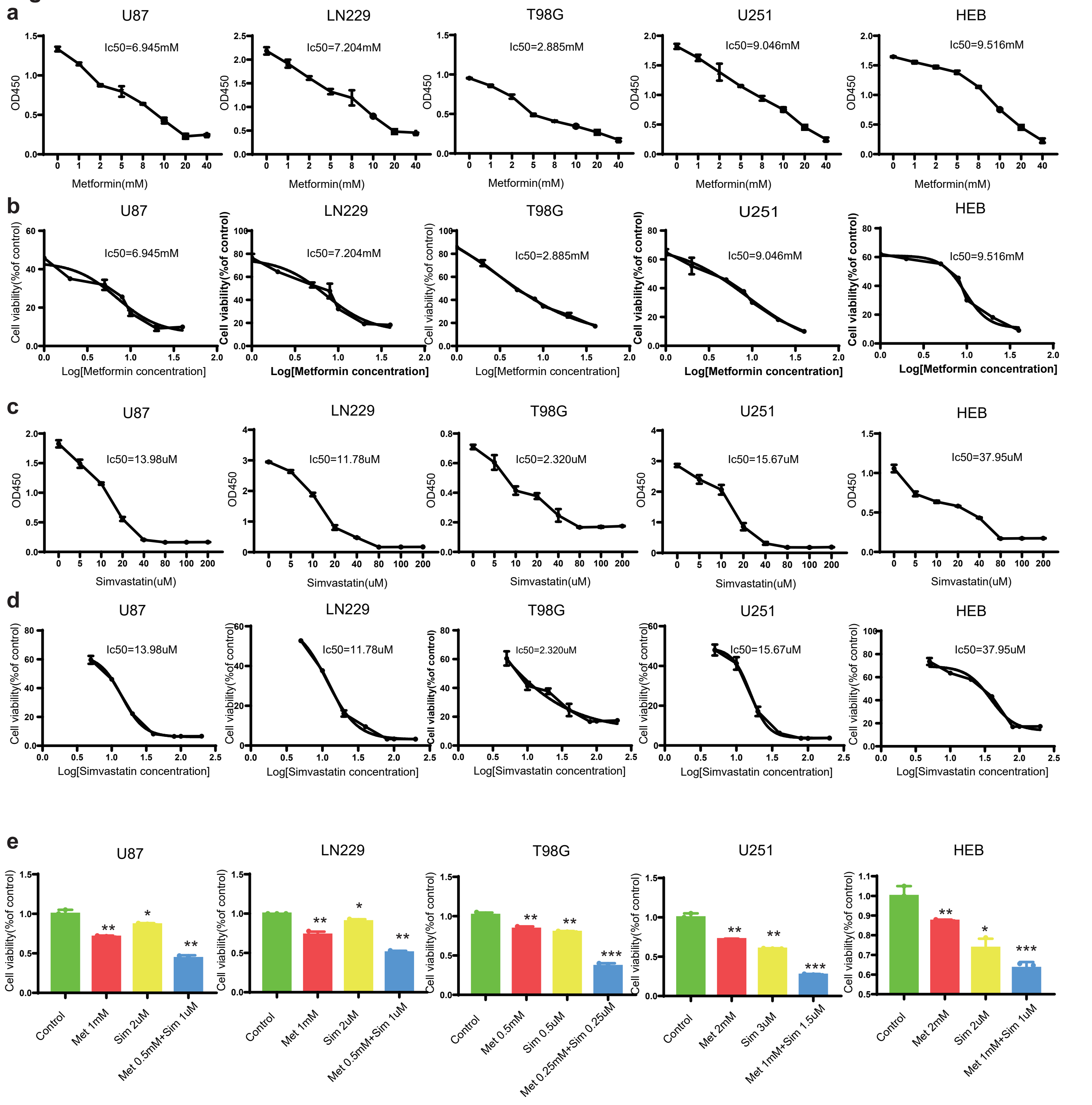
a
U87
Ic50=6.945mM
LN229
Ic50=7.204mM
T98G
Ic50=2.885mM
U251
Ic50=9.046mM
HEB
Ic50=9.516mM
OD450
Metformin(mM)
b
Cell viability(%of control)
Log[Metformin concentration]
c
Ic50=13.98uM
Ic50=11.78uM
Ic50=2.320uM
Ic50=15.67uM
Ic50=37.95uM
Simvastatin(uM)
d
Log[Simvastatin concentration]
e
Control
Met 1mM
Sim 2uM
Met 0.5mM+Sim 1uM
Met 0.5mM
Sim 0.5uM
Met 0.25mM+Sim 0.25uM
Met 2mM
Sim 3uM
Met 1mM+Sim 1.5uM
Met 1mM+Sim 1uM

Figure S3

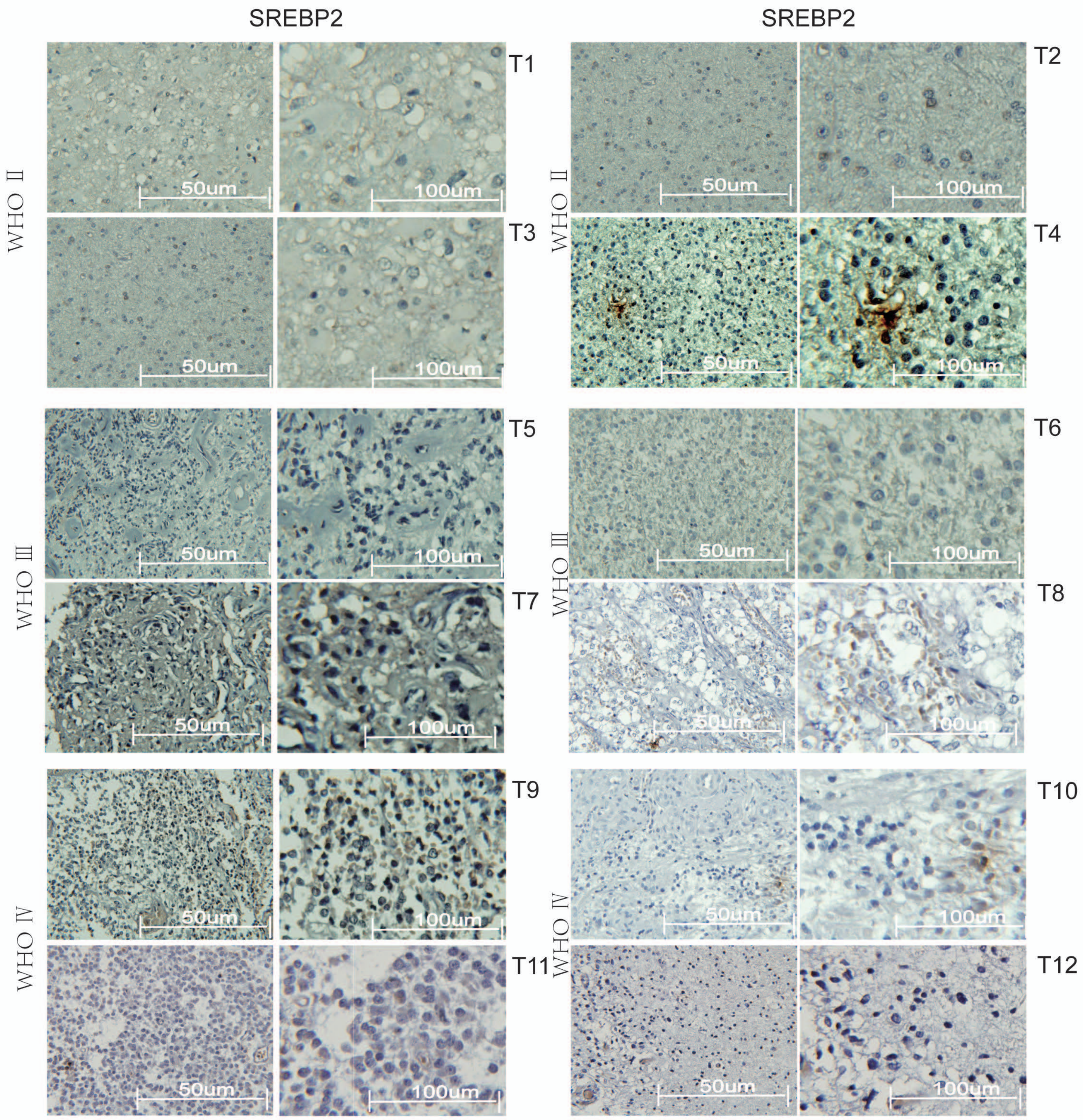
SREBP2
SREBP2
WHO Ⅱ
WHO Ⅱ
WHO Ⅲ
WHO Ⅲ
WHO Ⅳ
WHO Ⅳ
T1
T2
T3
T4
T5
T6
T7
T8
T9
T10
T11
T12
50um
100um

Figure S4

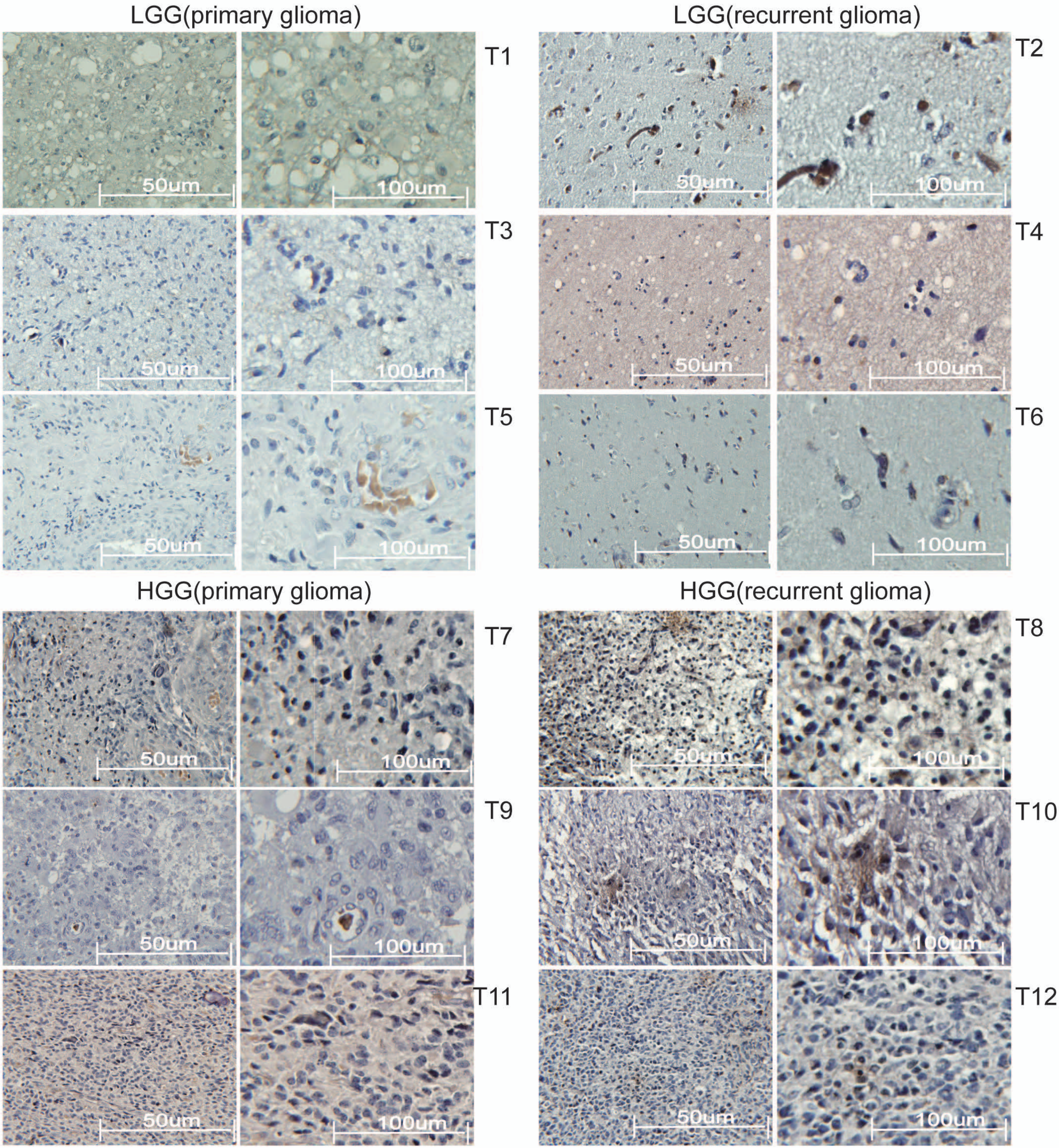
LGG(primary glioma)
LGG(recurrent glioma)
T1
T2
T3
T4
T5
T6
HGG(primary glioma)
HGG(recurrent glioma)
T7
T8
T9
T10
T11
T12
50um
100um

HEB Identification Report

细胞遗传质量鉴定检测

Cell Line Authentication Service

STR 基因型检测报告

送检单位：上海澳音生物科技有限公司
检品名称：细胞系
委托单位：上海翼和应用生物技术有限公司
报告日期：2023-05-17

上海翼和应用生物技术有限公司 联系地址：上海市松江区龙腾路 1015 弄中星创意园 2 号 502 室
网址：http://www.biowing.com.cn/邮箱：market@biowing.com.cn 服务热线：021-33559491

报 告 说 明

1. 本报告只对送检的来样负责。
2. 检验报告上的检验结果和检验单位名称，未经同意不得用于广告、评优及商业宣传。
3. 对本报告有异议，请于收到报告之日起十五日内以书面方式提出，逾期不予受理。
4. 对纸质检验报告涂改、增删，或未加盖检验单位印章的复印件均无效。

上海翼和应用生物技术有限公司 联系地址：上海市松江区龙腾路 1015 弄中星创意园 2 号 502 室
网址：http://www.biowing.com.cn/邮箱：market@biowing.com.cn 服务热线：021-33559491

样 品 信 息

样品编号：

客户样本编号	公司编号
SAc0560	20230512-02

样品性状： 细胞系

检测项目： STR

送检单位： **上海澳音生物科技有限公司**

检测方法： 用 Axygen 的基因组抽提试剂盒提取 DNA，采用 21- STR 扩增方案扩增，在 ABI 3730XL 型遗传分析仪上对 STR 位点和性别基因 Amelogenin 进行检测。

检 测 结 果

(一) 检验基本情况

公司编号	多等位基因	匹配细胞系	细胞库	EV 值	匹配说明
20230512-02	无		DSMZ		无匹配

样本基因型检验结果

- 多等位基因指三等位及以上基因现象。
- 本次检测各细胞分型结果良好。

(二) 各样本描述

- 20230512-02：该株细胞 DNA 分型在细胞系检索中**没有找到匹配**的细胞系。本次检测在该细胞系中**发现多等位基因**。该细胞株未发现人源交叉污染，因数据库未登录 HEB 相关 STR 数据信息，无法显示匹配结果，若发表论文可提交该数据给杂志。

EV	Cell No.	Cell name	Locus names								
			D5S818	D13S317	D7S820	D16S539	VWA	TH01	AM	TPOX	CSF1PO
	Query (Your Cell)		7,9	9,11	11,11	11,12,13	16,18	7,9	X,Y	8,11	11,12

备注：待测细胞系与收录于 ATCC, DSMZ, JCRB 和 RIKEN 数据库的细胞系 STR 数据进行比对，未收录于以上细胞库的细胞系将无法匹配。

（三）样本分型结果

细胞的 STR 位点和 Amelogenin 位点的基因分型结果						
Loci	送检细胞 STR 信息			细胞库细胞 STR 信息		
	送检细胞名：SAc0560			细胞库细胞名:		
	Allele1	Allele2	Allele3	Allele1	Allele2	Allele3
D5S818	7	9				
D13S317	9	11				
D7S820	11	11				
D16S539	11	13				
VWA	16	18				
TH01	7	9				
AMEL	X	Y				
TPOX	8	11				
CSF1PO	11	12				
D12S391	17	20				
FGA	19	21				
D2S1338	19	24				
D21S11	29	30				
D18S51	15	17				
D8S1179	12	13				
D3S1358	15	18				
D6S1043	10	14				
PENTAE	9	13				
D19S433	15	15.2				
PENTAD	9	9				
D1S1656	16	18				

上海翼和应用生物技术有限公司 联系地址：上海市松江区龙腾路 1015 弄中星创意园 2 号 502 室
网址：http://www.biowing.com.cn/邮箱：market@biowing.com.cn 服务热线：021-33559491

其他说明

(一) 分型方案及位点分布

	方案 1	方案 2	方案 3	方案 4
1	D3S1358	D8S1179	D19S433	AMEL
2	VWA	D21S11	TH01	D1S1656
3	D7S820	D16S539	D13S317	D5S818
4	CSF1PO	D2S1338	TPOX	D12S391
5	PENTAE	PENTAD	D18S51	FGA
6			D6S1043	

实验方案及位点

(二) STR 数据库比对

本公司采用 DSMZ tools 进行细胞系比对，其中包含来自于 ATCC, DSMZ, JCRB 和 RIKEN 数据库的 2455 个细胞系 STR 数据。如果待检测细胞未收录于以上细胞库或这是自行建立的新细胞系将无法进行比对，用户需根据细胞分型结果自行与其他数据库进行比对。

(三) 文献引用参考

1．Authentication testing of HEK 293T and HeLa cell lines have been performed by Shanghai Biowing Applied Biotechnology Co.,Ltd via STR profiling. STR profiles match the standards recommended for HEK 293T and HeLa cell lines authentication

2．AGS, NCI-N87, HGC-27 and HEK293 were STR-authenticated on Dec. 8, 2015 by Shanghai Biowing Applied Biotechnology Co. LTD, Shanghai, China

主要实验人员：何秀川

复核人: 张晨茜

负责人： 王敏

签发日期：2023-05-17

上海翼和应用生物技术有限公司 联系地址：上海市松江区龙腾路 1015 弄中星创意园 2 号 502 室
网址：http://www.biowing.com.cn/邮箱：market@biowing.com.cn 服务热线：021-33559491

Applied Biosystems
GeneMapper ID v3.2

20230512ren

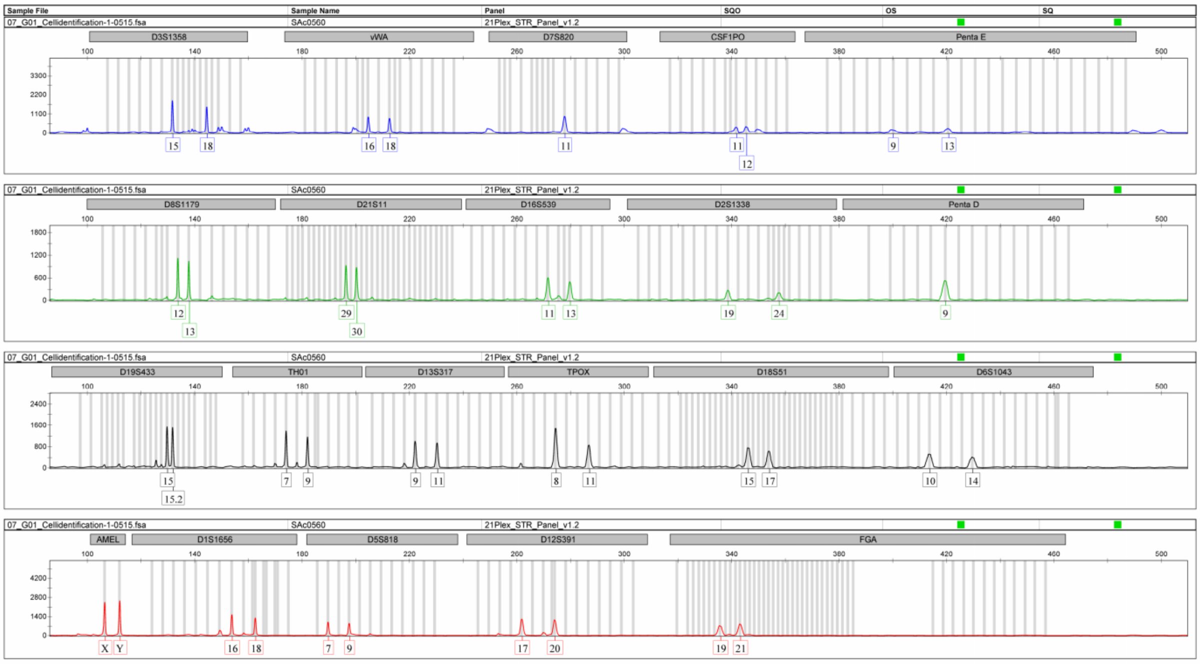

Tue May 16,2023 09:45AM, CST

Printed by: gmid

Page 1 of 1

T98G Identification Report

细胞遗传质量鉴定检测

Cell Line Authentication Service

STR 基因型检测报告

送检单位：赛百慷

检品名称：细胞系

委托单位：上海翼和应用生物技术有限公司

报告日期：2022-06-14

报 告 说 明

1. 本报告只对送检的来样负责。
2. 检验报告上的检验结果和检验单位名称，未经同意不得用于广告、评优及商业宣传。
3. 对本报告有异议，请于收到报告之日起十五日内以书面方式提出，逾期不予受理。
4. 对纸质检验报告涂改、增删，或未加盖检验单位印章的复印件均无效。

样 品 信 息

样品编号：

客户样本编号	公司编号
T98G	20220607-08

样品数量： 1

样品性状： 细胞系

检测项目： STR

送检单位： **赛百慷**

检测方法： 用 Axygen 的基因组抽提试剂盒提取 DNA，采用 21- STR 扩增方案扩增，在 ABI 3730XL 型遗传分析仪上对 STR 位点和性别基因 Amelogenin 进行检测。

检 测 结 果

(一) 检验基本情况

公司编号	多等位基因	匹配细胞系	细胞库	EV 值	匹配说明
20220607-08	无	T98G [T98-G]	DSMZ	1.0	完全匹配

样本基因型检验结果

- 多等位基因指三等位及以上基因现象。
- 本次检测各细胞分型结果良好。

(二) 各样本描述

- 20220607-08：该株细胞 DNA 分型在细胞系检索中找到**完全匹配**的细胞系，DSMZ 数据库显示细胞名为 **T98G [T98-G]**，细胞号对应 **CRL-1690**。本次检测在该细胞系中**没有发现多等位基因**。

备注：待测细胞系与收录于 ATCC, DSMZ, JCRB 和 RIKEN 数据库的细胞系 STR 数据进行比对，未收录于以上细胞库的细胞系将无法匹配。

EV	Cell No.	Cell name	Locus names								
			D5S818	D13S317	D7S820	D16S539	VWA	TH01	AM	TPOX	CSF1PO
	Query (Your Cell)		10,12	13,13	9,10	13,13	17,20	7,9.3	X,Y	8,8	10,12
1.0(36/36)	CRL-1690	T98G [T98-G]	['10', '12']	['13', '13']	['9', '10']	['13', '13']	['17', '20']	['7', '9.3']	['X', 'Y']	['8', '8']	['10', '12']
1.0(36/36)	IFO50303	T98G	['10', '12']	['13', '13']	['9', '10']	['13', '13']	['17', '20']	['7', '9.3']	['X', 'Y']	['8', '8']	['10', '12']
1.0(36/36)	JCRB9041	T98G	['10', '12']	['13', '13']	['9', '10']	['13', '13']	['17', '20']	['7', '9.3']	['X', 'Y']	['8', '8']	['10', '12']
1.0(36/36)	RCB1954	T98G	['10', '12']	['13', '13']	['9', '10']	['13', '13']	['17', '20']	['7', '9.3']	['X', 'Y']	['8', '8']	['10', '12']
0.94(34/36)	CVCL_0556Best	T98G	['10', '12']	['13', '13']	['9', '9']	['13', '13']	['17', '20']	['7', '9.3']	['X', 'Y']	['8', '8']	['10', '12']

（三）样本分型结果

细胞 20220607-08 的 STR 位点和 Amelogenin 位点的基因分型结果						
Loci	送检细胞 STR 信息			细胞库细胞 STR 信息		
	送检细胞名：T98G			细胞库细胞名：T98G [T98-G]		
	Allele1	Allele2	Allele3	Allele1	Allele2	Allele3
D5S818	10	12		10	12	
D13S317	13	13		13	13	
D7S820	9	10		9	10	
D16S539	13	13		13	13	
VWA	17	20		17	20	
TH01	7	9.3		7	9.3	
AMEL	X	Y		X	Y	
TPOX	8	8		8	8	
CSF1PO	10	12		10	12	
D12S391	17	18				
FGA	21	21				
D2S1338	19	24				
D21S11	28	32.2				
D18S51	13	16				
D8S1179	13	14				
D3S1358	16	16				
D6S1043	14	14				
PENTAE	16	16				
D19S433	12	12				
PENTAD	10	11				
D1S1656	14	17				

其他说明

(一) 分型方案及位点分布

	方案 1	方案 2	方案 3	方案 4
1	D3S1358	D8S1179	D19S433	AMEL
2	VWA	D21S11	TH01	D1S1656
3	D7S820	D16S539	D13S317	D5S818
4	CSF1PO	D2S1338	TPOX	D12S391
5	PENTAE	PENTAD	D18S51	FGA
6			D6S1043	

实验方案及位点

(二) STR 数据库比对

本公司采用 DSMZ tools 进行细胞系比对，其中包含来自于 ATCC, DSMZ, JCRB 和 RIKEN 数据库的 2455 个细胞系 STR 数据。如果待检测细胞未收录于以上细胞库或这是自行建立的新细胞系将无法进行比对，用户需根据细胞分型结果自行与其他数据库进行比对。

(三) 文献引用参考

1．Authentication testing of HEK 293T and HeLa cell lines have been performed by Shanghai Biowing Applied Biotechnology Co.,Ltd via STR profiling. STR profiles match the standards recommended for HEK 293T and HeLa cell lines authentication

2．AGS, NCI-N87, HGC-27 and HEK293 were STR-authenticated on Dec. 8, 2015 by Shanghai Biowing Applied Biotechnology Co. LTD, Shanghai, China

主要实验人员：张佳男

复核人: 钱宁

负责人： 白杨

签发日期：2022-06-14

U87 Identification Report

细胞遗传质量鉴定检测

Cell Line Authentication Service

STR 基因型检测报告

送检单位：赛百慷
检品名称：细胞系
委托单位：上海翼和应用生物技术有限公司
报告日期：2022 年 06 月 14 日

上海翼和应用生物技术有限公司 联系地址：上海市漕河泾开发区桂平路 471 号桂果园 8 号楼 4F 室
网址：http://www.biowing.com.cn/邮箱：biowing@vip.163.com 服务热线：021-33559491

报 告 说 明

1. 本报告只对送检的来样负责。
2. 检验报告上的检验结果和检验单位名称，未经同意不得用于广告、评优及商业宣传。
3. 对本报告有异议，请于收到报告之日起十五日内以书面方式提出，逾期不予受理。
4. 对纸质检验报告涂改、增删，或未加盖检验单位印章的复印件均无效。

上海翼和应用生物技术有限公司　联系地址：上海市漕河泾开发区桂平路 471 号桂果园 8 号楼 4F 室
网址：http://www.biowing.com.cn/邮箱：biowing@vip.163.com　服务热线：021-33559491

样 品 信 息

样品编号：

客户样本编号	公司编号
U87MG	20220607-12

样品数量： 1

样品性状： 细胞系

检测项目： STR

送检单位： 赛百慷

检测方法： 用 Axygen 的基因组抽提试剂盒提取 DNA，采用 20- STR 扩增方案扩增，在 ABI 3730XL 型遗传分析仪上对 STR 位点和性别基因 Amelogenin 进行检测。

上海翼和应用生物技术有限公司 联系地址：上海市漕河泾开发区桂平路 471 号桂果园 8 号楼 4F 室
网址：http://www.biowing.com.cn/邮箱：biowing@vip.163.com 服务热线：021-33559491

检 测 结 果

(一) 检验基本情况

	多等位基因	匹配细胞系	细胞库	EV 值	匹配说明
20220607-12	有	U-87MG	DSMZ	1	完全匹配

样本基因型检验结果

- 多等位基因指三等位及以上基因现象。
- 本次检测各细胞分型结果良好。

(二) 各样本描述

20220607-12：该株细胞 DNA 分型在细胞系检索中找到完全匹配的细胞系，DSMZ 数据库显示细胞名为 **U-87MG**，细胞号对应 **HTB-14**。本次检测在该细胞系中发现多等位基因。

备注：待测细胞系与收录于 ATCC, DSMZ, JCRB 和 RIKEN 数据库的细胞系 STR 数据进行比对，未收录于以上细胞库的细胞系将无法匹配。

EV	Cell No.	Cell name	Locus names									Figures
			D5S818	D13S317	D7S820	D16S539	VWA	TH01	AM	TPOX	CSF1PO	
	Query (Your Cell)		11,12	8,11	8,9	12,12	15,17	9.3,9.3	x,x	8,8	10,11	
1.00(36/36)	HTB-14	U-87MG	11,12	8,11	8,9	12,12	15,17	9.3,9.3	X,X	8,8	10,11	-
0.72(26/36)	731	CAKI-1	11,12	11,11	8,12	12,12	15,17	6,8	X,X	8,11	10,11	-
0.72(26/36)	749	U-CH2	10,11	11,11	8,12	12,12	17,17	9.3,9.3	X,X	8,8	11,12	-
0.72(26/36)	CRL-5842	NCI-H774 [H774]	11,11	8,8	9,11	12,12	15,17	6,9.3	X,X	8,8	10,10	-
0.72(26/36)	CRL-5910	NCI-H1994 [H1994]	10,11	11,11	9,11	12,12	15,19	7,9.3	X,X	8,8	10,11	-
0.72(26/36)	CRL-7064	Hs 94.T	11,12	12,13	8,9	9,12	17,18	7,9.3	X,X	8,8	10,11	-

上海翼和应用生物技术有限公司 联系地址：上海市漕河泾开发区桂平路 471 号桂果园 8 号楼 4F 室
网址：http://www.biowing.com.cn/邮箱：biowing@vip.163.com 服务热线：021-33559491

（三）样本分型结果

细胞 20220607-12 的 STR 位点和 Amelogenin 位点的基因分型结果						
Loci	送检细胞 STR 信息			细胞库细胞 STR 信息		
	送检细胞名：U87MG			细胞库细胞名：U-87MG		
	Allele1	Allele2	Allele3	Allele1	Allele2	Allele3
D5S818	11	12	13	11	12	
D13S317	8	11		8	11	
D7S820	8	9		8	9	
D16S539	12	12		12	12	
VWA	15	17		15	17	
TH01	9.3	9.3		9.3	9.3	
AMEL	X	X		X	X	
TPOX	8	8		8	8	
CSF1PO	10	11		10	11	
D12S391	18	21				
FGA	18	24				
D2S1338	20	23				
D21S11	28	32.2				
D18S51	13	13				
D8S1179	10	11				
D3S1358	16	17				
D6S1043	11	18				
PENTAE	7	14				
D19S433	15	15.2				
PENTAD	9	14				

上海翼和应用生物技术有限公司 联系地址：上海市漕河泾开发区桂平路 471 号桂果园 8 号楼 4F 室
网址：http://www.biowing.com.cn/邮箱：biowing@vip.163.com 服务热线：021-33559491

其 他 说 明

(一) 分型方案及位点分布

	方案 1	方案 2	方案 3	方案 4
1	TH01	TPOX	D3S1358	AMEL
2	D12S391	VWA	D13S317	D5S818
3	D7S820	D8S1179	D6S1043	D2S1338
4	CSF1PO	PENTAD	D16S539	D21S11
5	FGA		D19S433	D18S51
6	PENTAE			

实验方案及位点

(二) STR 数据库比对

本公司采用 DSMZ tools 进行细胞系比对，其中包含来自于 ATCC, DSMZ, JCRB 和 RIKEN 数据库的 2455 个细胞系 STR 数据。如果待检测细胞未收录于以上细胞库或这是自行建立的新细胞系将无法进行比对，用户需根据细胞分型结果自行与其他数据库进行比对。

(三) 文献引用参考

1．Authentication testing of HEK 293T and HeLa cell lines have been performed by Shanghai Biowing Applied Biotechnology Co.,Ltd via STR profiling. STR profiles match the standards recommended for HEK 293T and HeLa cell lines authentication

2．AGS, NCI-N87, HGC-27 and HEK293 were STR-authenticated on Dec. 8, 2015 by Shanghai Biowing Applied Biotechnology Co. LTD, Shanghai, China

主要实验人员：张佳男

复核人: 龚礼瑾

负责人： 陈轶群

签发日期：2022 年 06 月 14 日

上海翼和应用生物技术有限公司 联系地址：上海市漕河泾开发区桂平路 471 号桂果园 8 号楼 4F 室
网址：http://www.biowing.com.cn/邮箱：biowing@vip.163.com 服务热线：021-33559491
