# Supplementary material for: Combined metformin and simvastatin therapy inhibits SREBP2 maturation and alters energy metabolism in glioma
Source: Cell Death Dis. 2024 Nov 9;15(11):809. doi: 10.1038/s41419-024-07169-5 (PMC11550444; doi:10.1038/s41419-024-07169-5)

Fig.2c

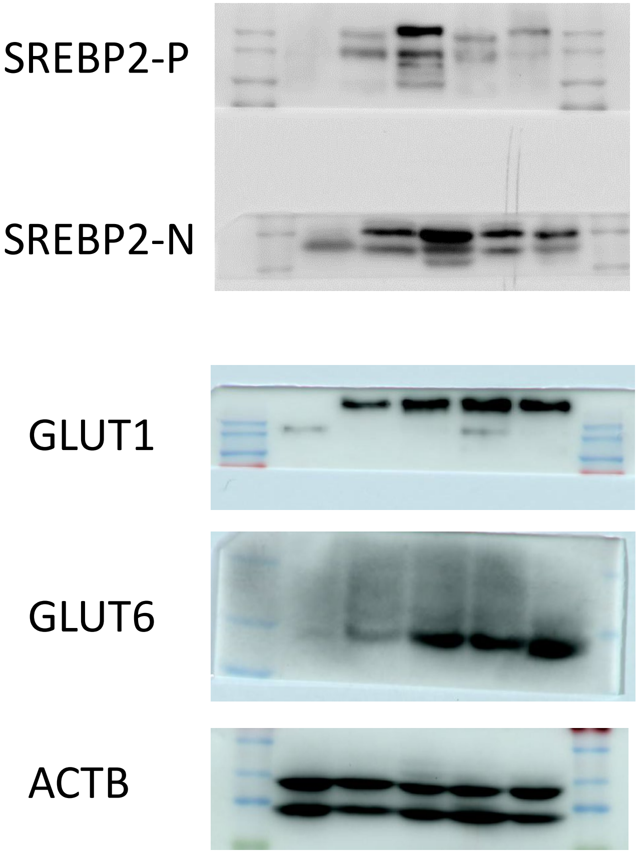

SREBP2-P
SREBP2-N
GLUT1
GLUT6
ACTB

Fig.2d

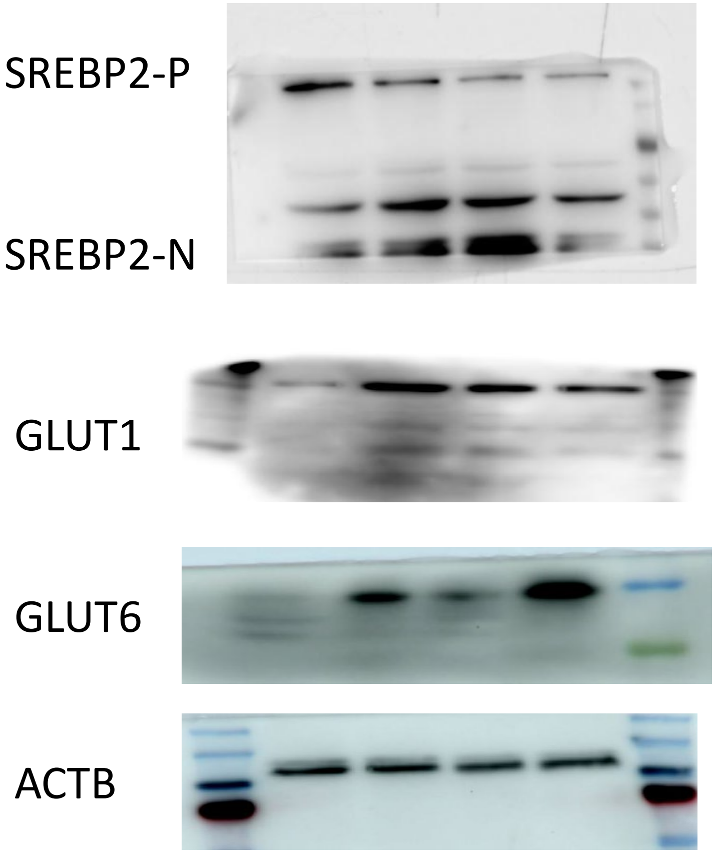

SREBP2-P
SREBP2-N
GLUT1
GLUT6
ACTB

Fig.3a

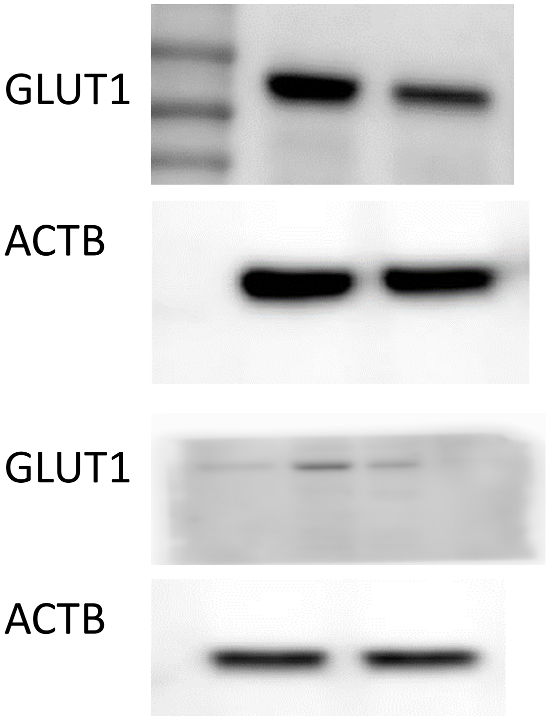

Fig.3b

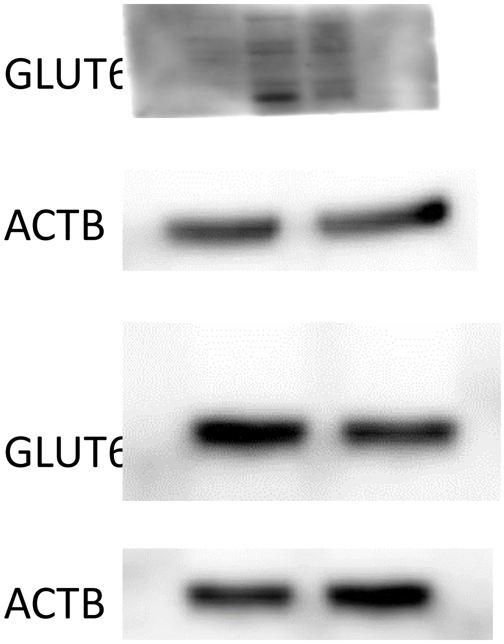

Fig.4F

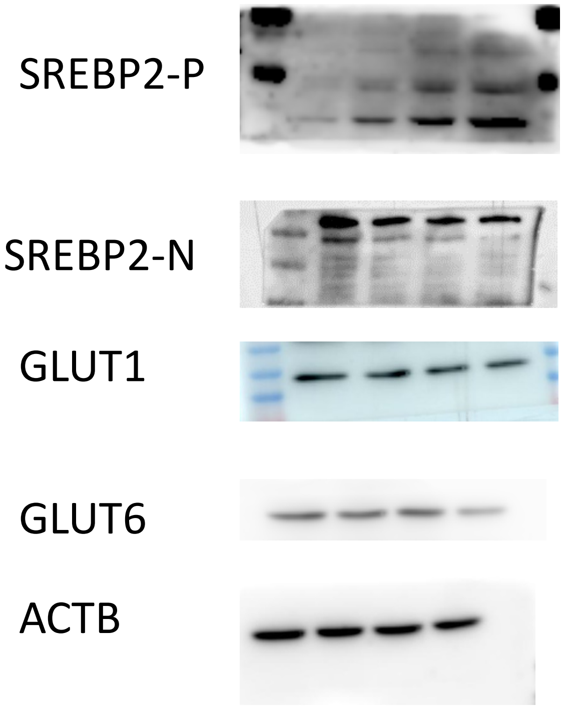

Fig.4H

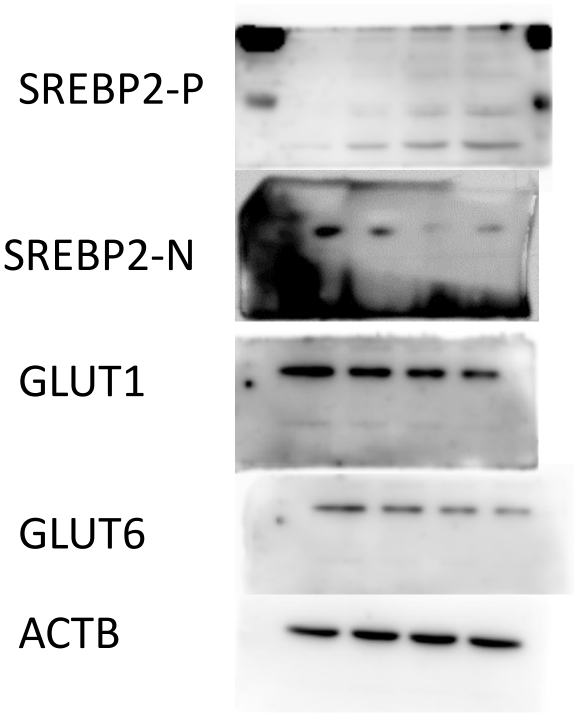

Fig.4J

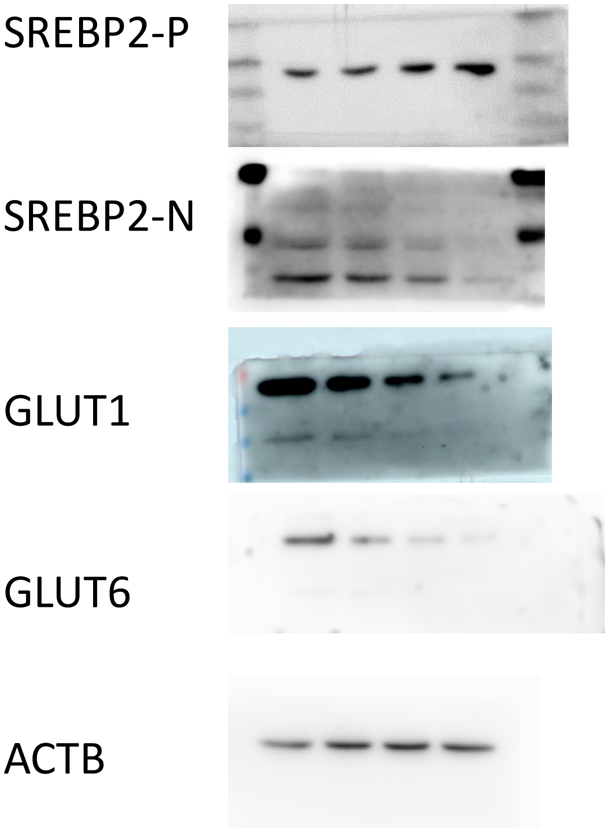

Fig.5C

SREBP2-P

SREBP2-N

SCAP

MBTPS1

ACTB

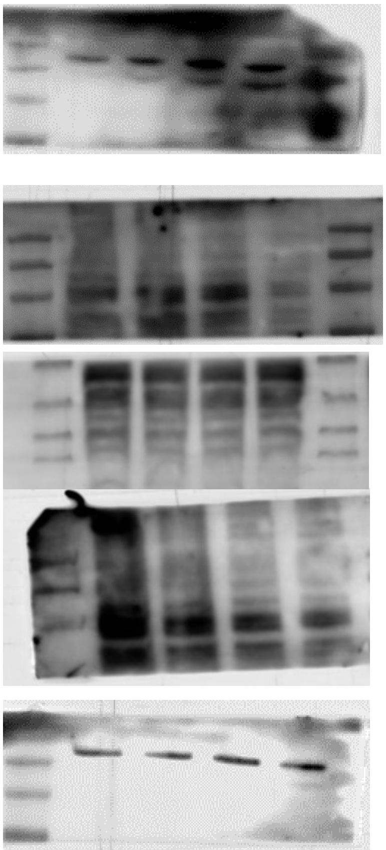

Fig.5C

SREBP2-P

SREBP2-N

SCAP

MBTPS1

ACTB

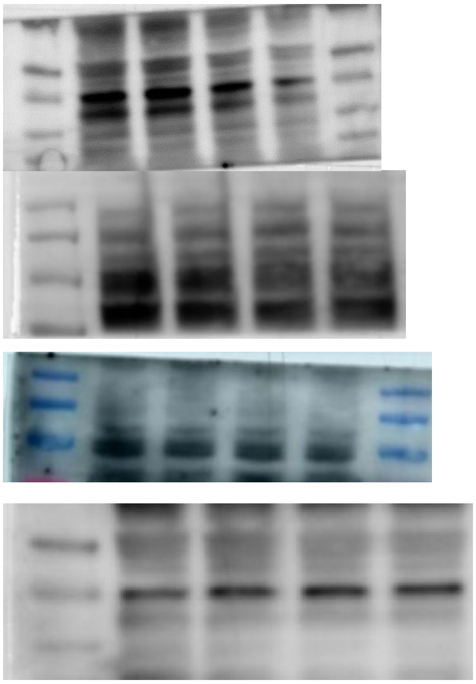

Fig.5C

SREBP2-P

SREBP2-N

SCAP

MBTPS1

ACTB

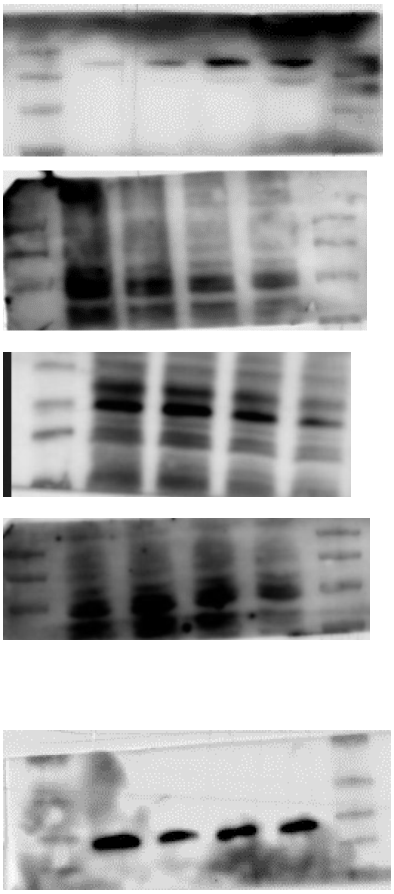

Fig.5C

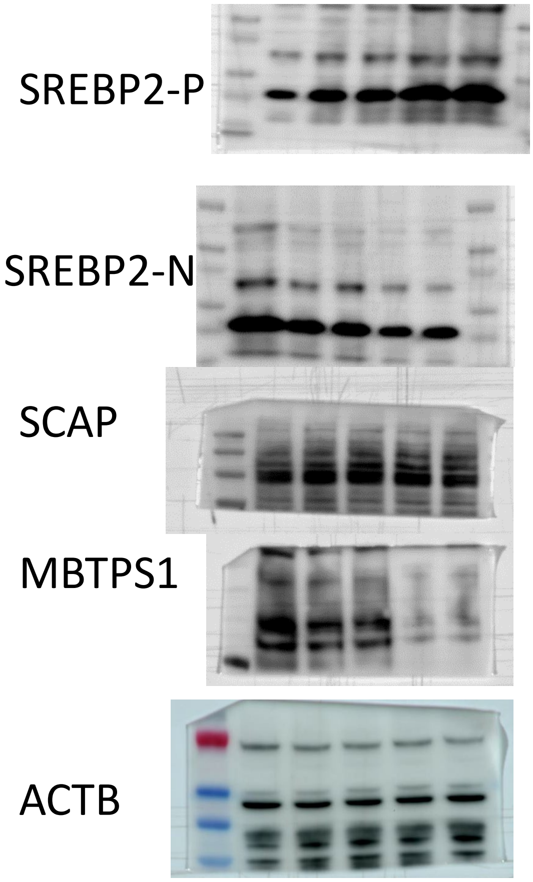

Fig.5C

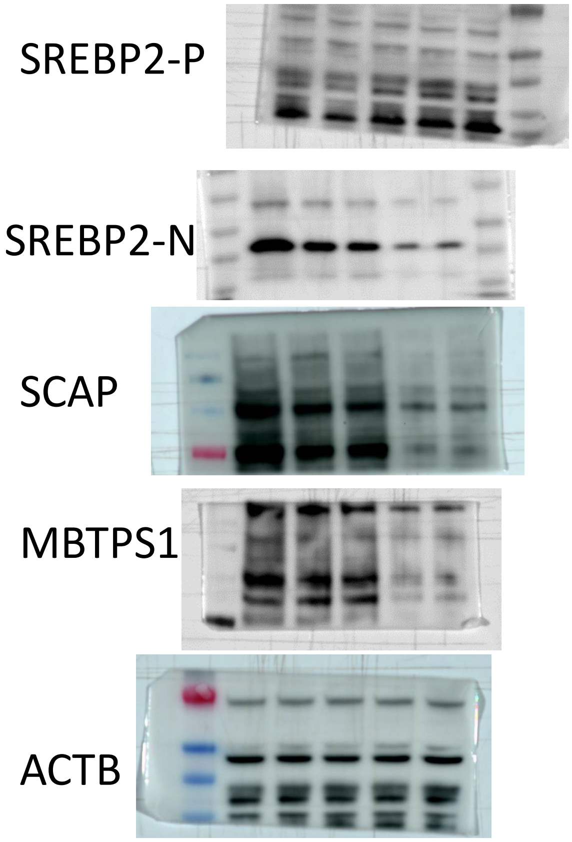

Fig.5C

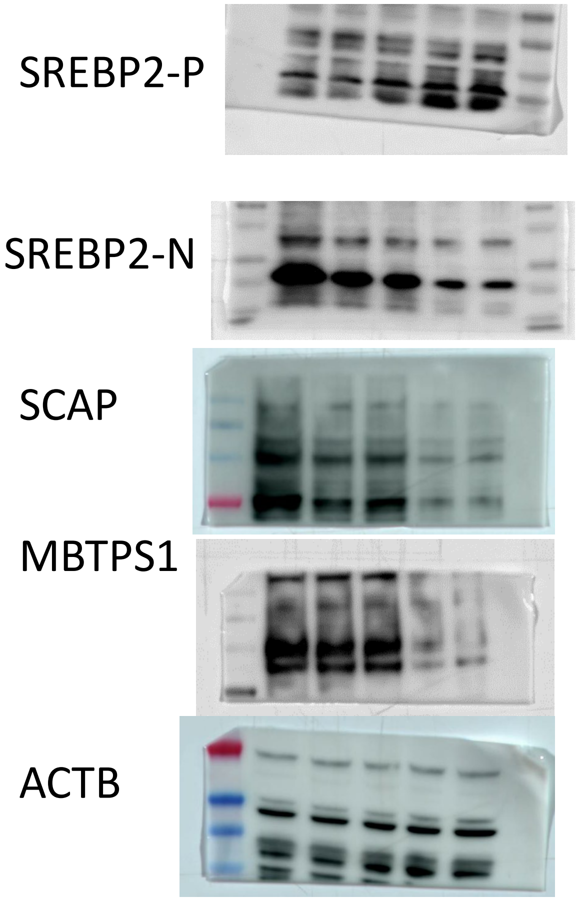

Fig.5D

SREBP2-P

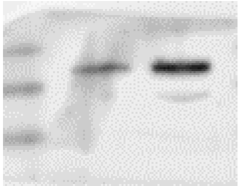

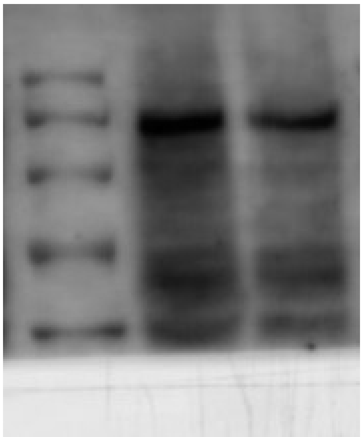

SREBP2-P

SREBP2-N

SCAP

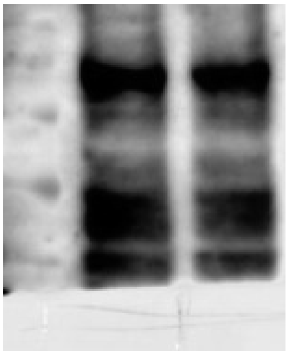

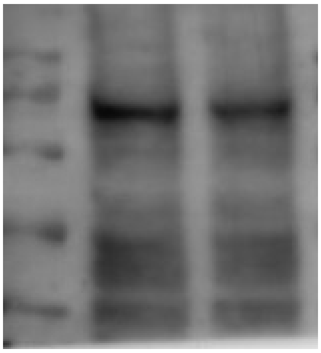

SCAP

ACTB

SREBP2-P

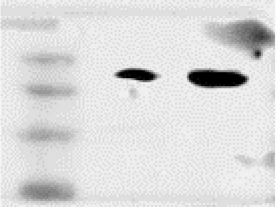

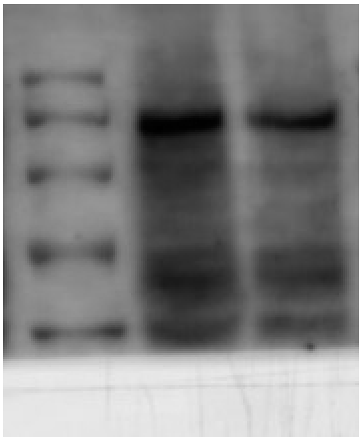

SREBP2-P

SREBP2-N

SCAP

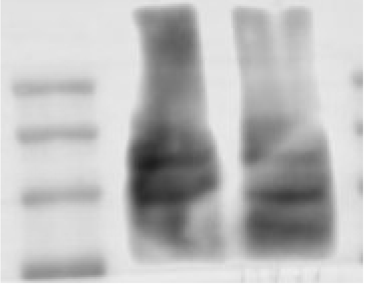

ACTB

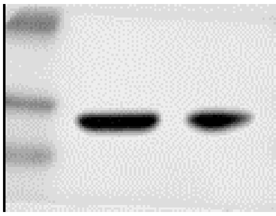

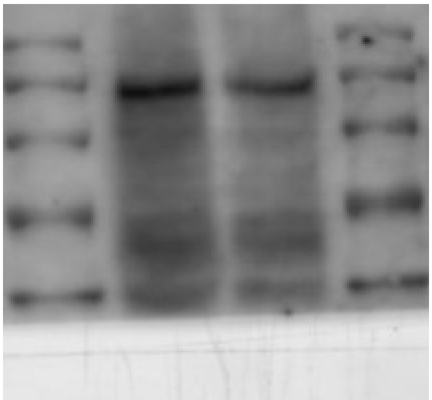

SCAP

Fig.5D

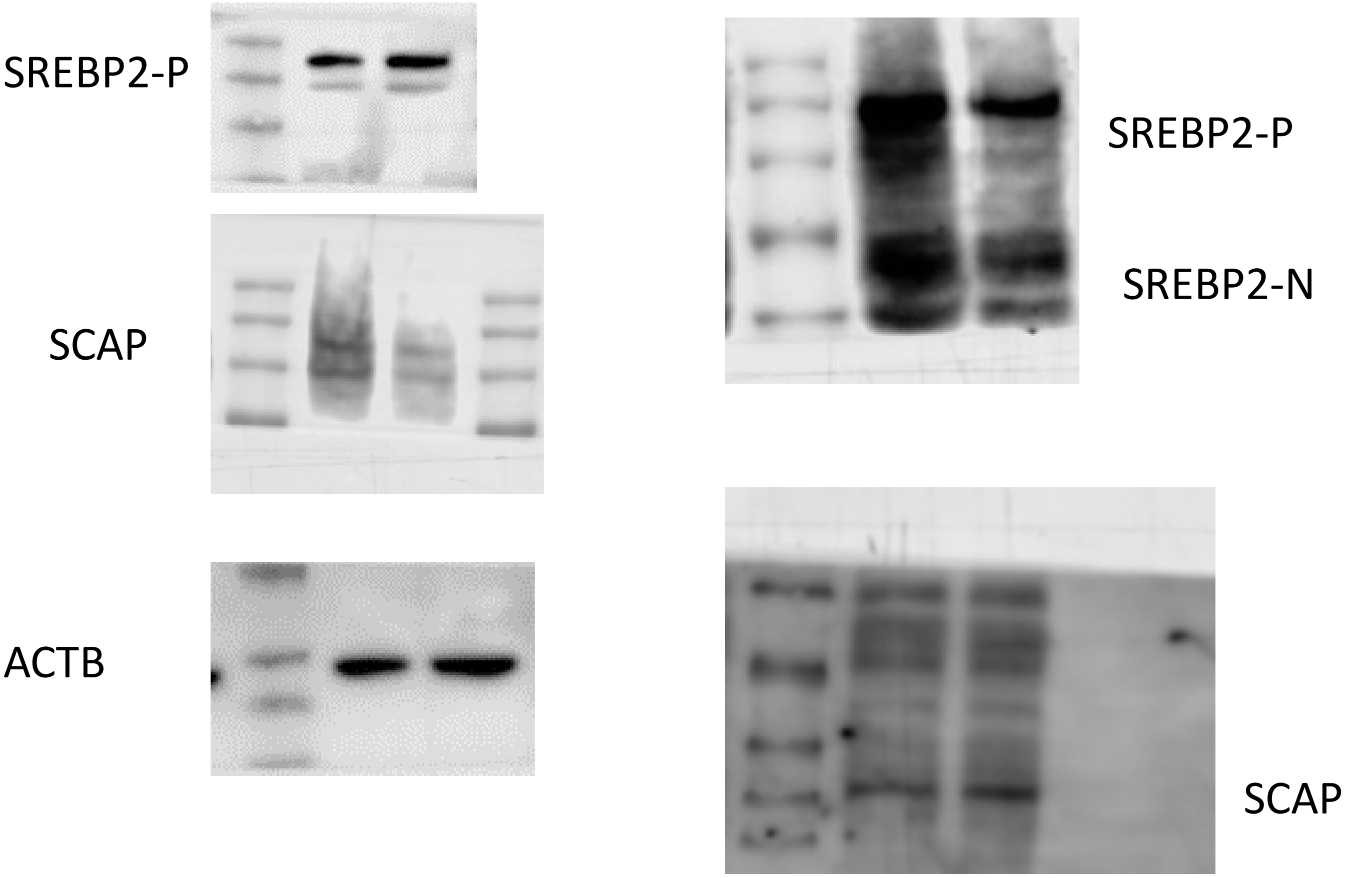

Fig.7e

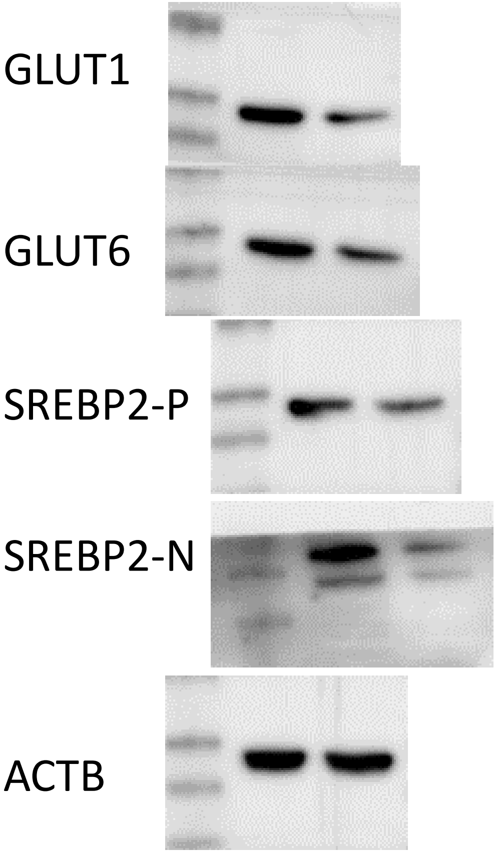

Fig.7f

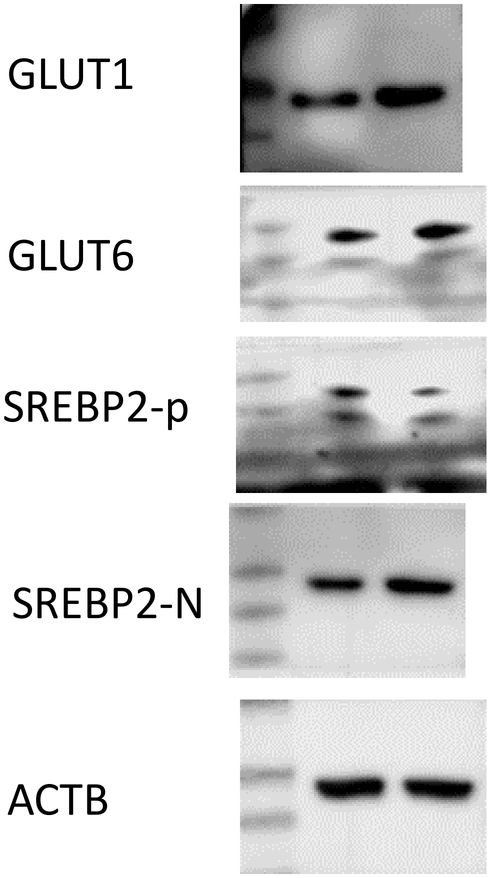

Fig.7g

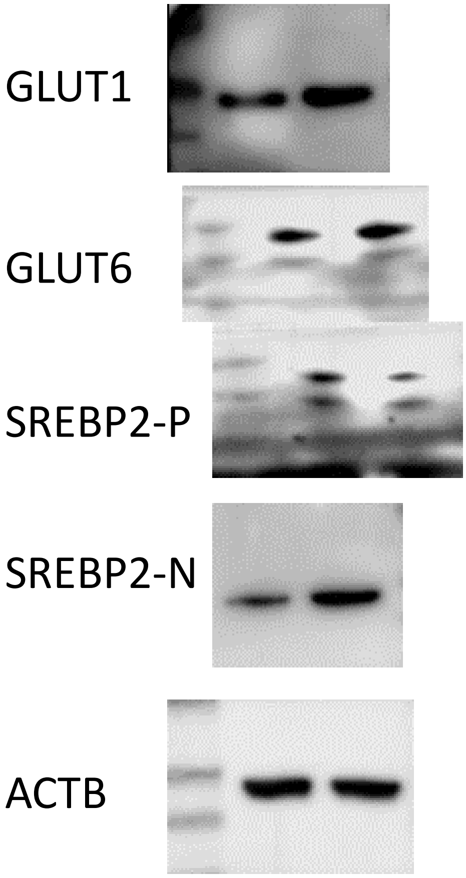

Fig.7h

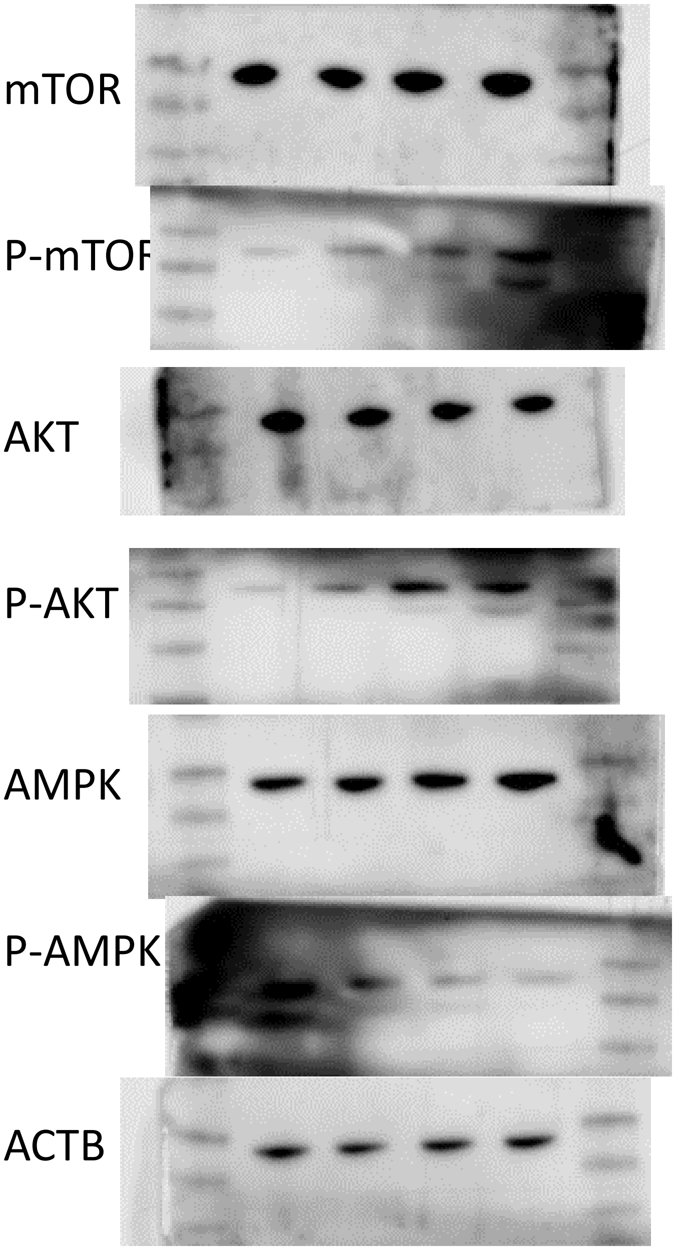

Fig.7i

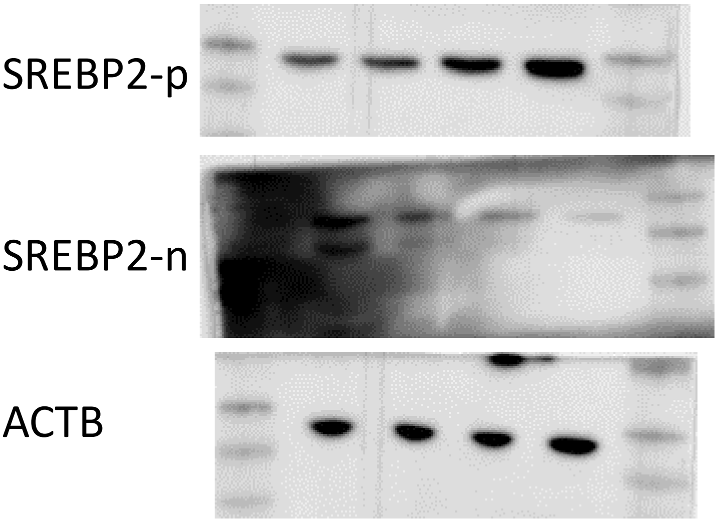

Supplement: Supplementary file 2 — original data files [file 41419_2024_7169_MOESM2_ESM.pdf]
